# Dissection of canopy layer-specific genetic control of leaf angle in *Sorghum bicolor* by RNA sequencing

**DOI:** 10.1186/s12864-021-08251-4

**Published:** 2022-02-03

**Authors:** Martha I. Natukunda, Maria B. Mantilla-Perez, Michelle A. Graham, Peng Liu, Maria G. Salas-Fernandez

**Affiliations:** 1 Department of Agronomy, Iowa State University, Ames, IA 50011 USA; 2 Present address: Bayer Crop Science, Chesterfield, MO USA; 3 Corn Insects and Crop Genetics Research, USDA-ARS, Ames, IA 50011 USA; 4 Department of Statistics, Iowa State University, Ames, IA 50011 USA

**Keywords:** Differentially expressed genes, Leaf angle, RNA sequencing, Smart canopy, Sorghum, Quantitative trait loci

## Abstract

**Background:**

Leaf angle is an important plant architecture trait, affecting plant density, light interception efficiency, photosynthetic rate, and yield. The “smart canopy” model proposes more vertical leaves in the top plant layers and more horizontal leaves in the lower canopy, maximizing conversion efficiency and photosynthesis. Sorghum leaf arrangement is opposite to that proposed in the “smart canopy” model, indicating the need for improvement. Although leaf angle quantitative trait loci (QTL) have been previously reported, only the *Dwarf3* (*Dw3*) auxin transporter gene, colocalizing with a major-effect QTL on chromosome 7, has been validated. Additionally, the genetic architecture of leaf angle across canopy layers remains to be elucidated.

**Results:**

This study characterized the canopy-layer specific transcriptome of five sorghum genotypes using RNA sequencing. A set of 284 differentially expressed genes for at least one layer comparison (FDR < 0.05) co-localized with 69 leaf angle QTL and were consistently identified across genotypes. These genes are involved in transmembrane transport, hormone regulation, oxidation-reduction process, response to stimuli, lipid metabolism, and photosynthesis. The most relevant eleven candidate genes for layer-specific angle modification include those homologous to genes controlling leaf angle in rice and maize or genes associated with cell size/expansion, shape, and cell number.

**Conclusions:**

Considering the predicted functions of candidate genes, their potential undesirable pleiotropic effects should be further investigated across tissues and developmental stages. Future validation of proposed candidates and exploitation through genetic engineering or gene editing strategies targeted to collar cells will bring researchers closer to the realization of a “smart canopy” sorghum.

**Supplementary Information:**

The online version contains supplementary material available at 10.1186/s12864-021-08251-4.

## Background

Improvement of plant architecture traits has enormous potential to increase grain and biomass yield [1–3]. Leaf angle, the inclination between the leaf blade midrib and the main stem [2, 4, 5], is one of the most important architecture traits in cereals as it affects planting density and photosynthetic capacity [6, 7]. Alteration of leaf angle to intercept a smaller fraction of light by the upper canopy has been proposed to allow more photosynthetically active radiation to reach the lower layers, ultimately increasing the conversion efficiency of overall intercepted light into biomass [6]. Subsequent modeling studies proposed an ideotype called “smart canopy,” which includes better leaf arrangement, improved catalytic activity and specificity of Rubisco, and changes in reaction center numbers and antenna sizes throughout the canopy [7]. The proposed leaf arrangement is characterized by more vertical upper leaves, with gradually increasing angles towards the middle and lower canopy layers [6, 7]. This architecture would increase conversion efficiency by minimizing light saturation at the upper canopy and increase the carbon fixation capacity of lower leaves [6, 7].

Over the last two decades, several association analyses and linkage mapping studies were conducted to investigate the genetic control of leaf angle in sorghum, with most studies examining specific leaves but not the entire canopy [4, 5, 8–10]. The experimental design focused on individual leaves limited our understanding of leaf angle determination on a whole-plant basis, yet this is a critical aspect for developing photosynthetically efficient and higher yielding sorghum genotypes. A major-effect QTL controlling leaf angle was reported numerous times on chromosome 7 in a genomic region containing the *Dwarf 3* (*Dw3*) gene (*Sobic.007G163800*) [4, 5, 8, 9]. This initial 4.5 Mb chromosomal interval [8] was narrowed down to 3.9 Mb [4] and later to 1.67 Mb and 1.65 Mb [5, 11]. A tandem repeat in *Dw3*, a well-characterized auxin efflux transporter with pleiotropic effects on leaf angle and plant height, was identified and validated as the causal polymorphism [4, 9, 12, 13]. However, Zhao et al. [5] detected significant associations between leaf angle variation and markers in the region but not with *Dw3* itself, and thus, authors proposed other genes on the chromosome 7 interval could be controlling the trait as well.

In the first study to examine the genetic control of leaf angle throughout the sorghum canopy, Mantilla-Perez et al. [11] reported that leaf arrangement in diverse genotypes is opposite to that proposed in the “smart canopy” model both under controlled (greenhouse) and field conditions. Additionally, QTL and single nucleotide polymorphisms (SNP) controlling leaf angle at specific layers or the whole plant canopy were detected [11]. Although significant progress has been made to understand the genetic architecture of leaf angle in sorghum, specific genes controlling this complex trait at specific canopy layers have not been identified, except *Dw3*.

In cereals, leaf angle is determined by collar or lamina joint cell size [2, 5], and studies in rice have shown that more longitudinally elongated adaxial collar cells cause the leaf blade to bend away from the main stem, resulting in more horizontal leaves with larger angles [2, 14, 15]. Cell expansion is driven by increasing turgor pressure that results from water and cargo uptake, and cell wall relaxation is regulated by phytohormones such as auxin, brassinosteroids (BRs), and gibberellins (GA) [16, 17]. During cell expansion, nutrients such as potassium facilitate cell growth by providing the necessary osmotic potential for water uptake [18, 19]. In sorghum, auxin and BRs are the most studied phytohormones known to control leaf angle. Candidate genes from the BR biosynthesis and signaling pathways have been associated with leaf angle variation, with one of the genes being the BR transcription factor, *Brassinazole resistant 1-*-*BZR1* or *BRI1 EMS suppressor*-*BES1* (*BZR1/BES1*) [10]. Analysis of *Dw3* and *BZR1/BES1* expression patterns across the canopy using real-time quantitative PCR (RT-qPCR) revealed positive correlations between their expression and leaf angle from lower to upper canopy layers [11]. Considering this initial evidence of gene expression variation across canopy layers, the objective of this study was to dissect the layer-specific genetic control of leaf angle at the transcriptome level using RNA-seq and five genotypes contrasting for the target trait. This transcriptional profiling from collar tissue at the canopy level has facilitated the identification of candidate genes and gene networks that could be exploited for the development of improved plant architecture according to the “smart canopy” ideotype.

## Results

### Leaf angle phenotypes and canopy layer-specific transcriptome analysis

**Fig. 1:**
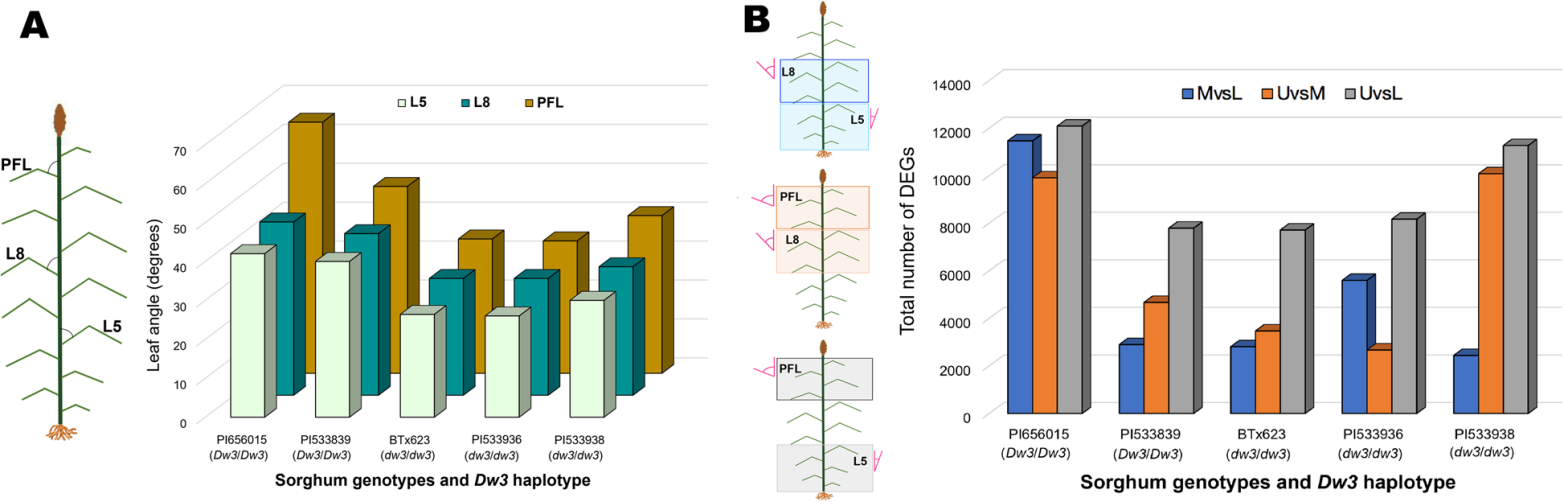
Leaf angle and differentially expressed genes (DEGs) across canopy layers. **A** Angle of leaves 5 (L5), 8 (L8), and pre-flag leaf (PFL) for the five sorghum genotypes representing the lower, middle, and upper canopy layers, respectively. **B** Total number of DEGs (FDR < 0.05) for the three canopy layer comparisons: middle vs. lower (MvsL-blue bars), upper vs. middle (UvsM-orange bars), upper vs. lower canopy (UvsL-grey bars)

Leaf angle gradually increased from lower to upper canopy layers for all five genotypes (Fig. 1A). This trend across the canopy is opposite to the proposed ideotype, suggesting the need to dissect the layer-specific genetic control of leaf angle. In agreement with the known role of *Dw3*, the two genotypes with the functional (*Dw3*/*Dw3*) haplotype (PI656015 and PI533839) had larger angles in all layers than those with the nonfunctional (*dw3*/*dw3*) haplotype (PI564163, PI533938 and PI533936) (Fig. 1A). Between-genotype variation in leaf angle magnitude was evident, with PI656015 consistently having the largest average angles at each layer (L5-42^o^, L8–44.5^o^, and PFL-64.5^o^) (Fig. 1A).

**Fig. 2:**
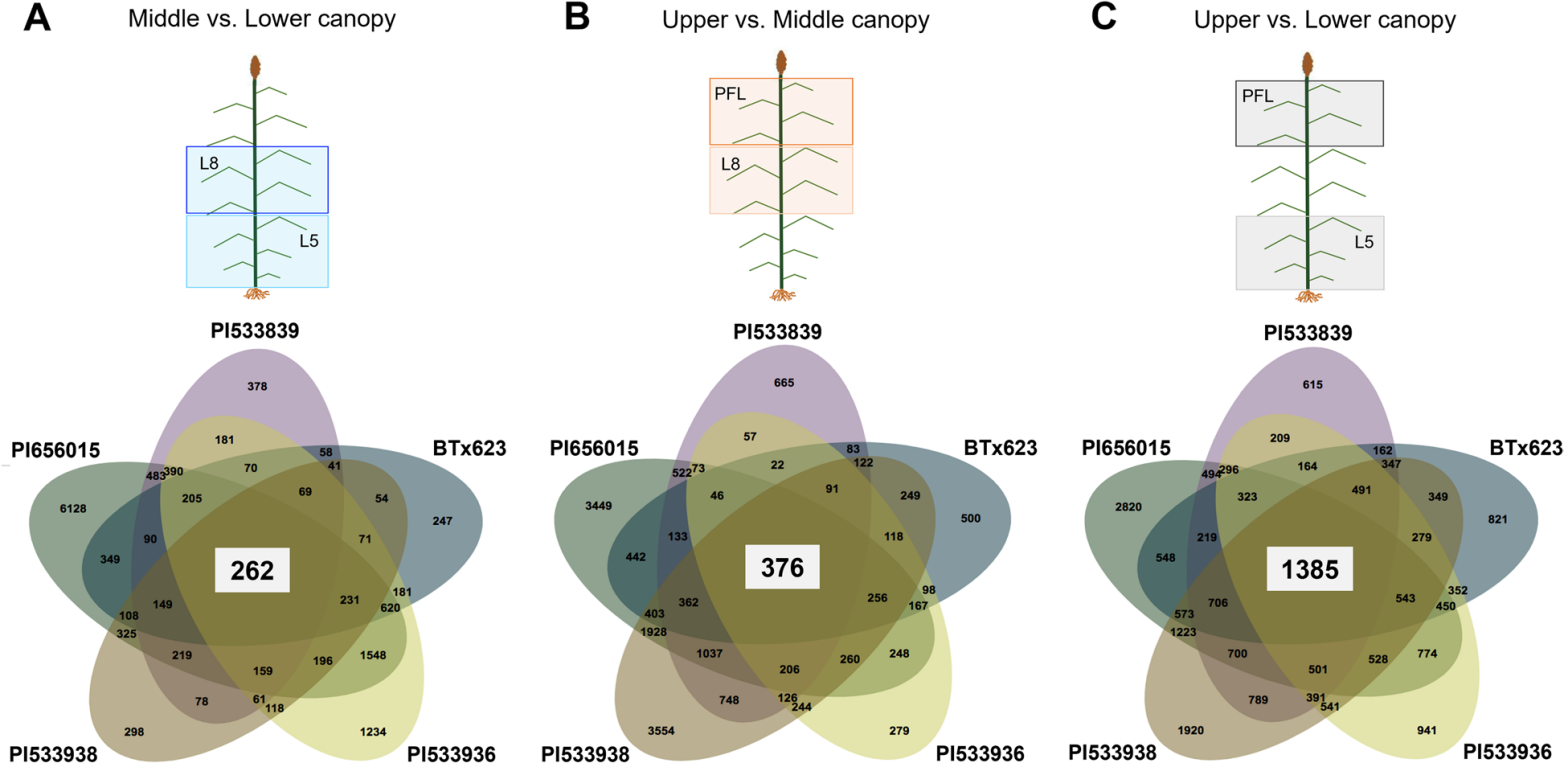
Common and unique DE gene sets among sorghum genotypes for the middle vs. lower (**A**), upper vs. middle (**B**), and the upper vs. lower (**C**) canopy layer comparisons

In total, 896,875,569,150-bp single-end raw reads were generated by RNA-seq, and after trimming, 880,209,941 reads were retained for subsequent analysis (Supplementary Table S1). The overall alignment rate to the sorghum reference genome v3 [20] ranged from 77.3 to 90.0% (Supplementary Table S1). The total number of DEGs (FDR < 0.05) varied by genotype and layer comparison between 2439 (for PI533839-MvsL) and 12,083 (for PI656015-UvsL) (Fig. 1B). Interestingly, the UvsL comparison rendered the highest number of DEG in all genotypes (range 12,083-7712) (Fig. 1B). Overlapping gene sets for specific canopy layer comparisons were examined to identify common DEGs across genotypes, which ranged from 1385 for UvsL to 262 for MvsL (Fig. 2).

### Differentially expressed genes co-localize with known leaf angle QTL

**Fig. 3:**
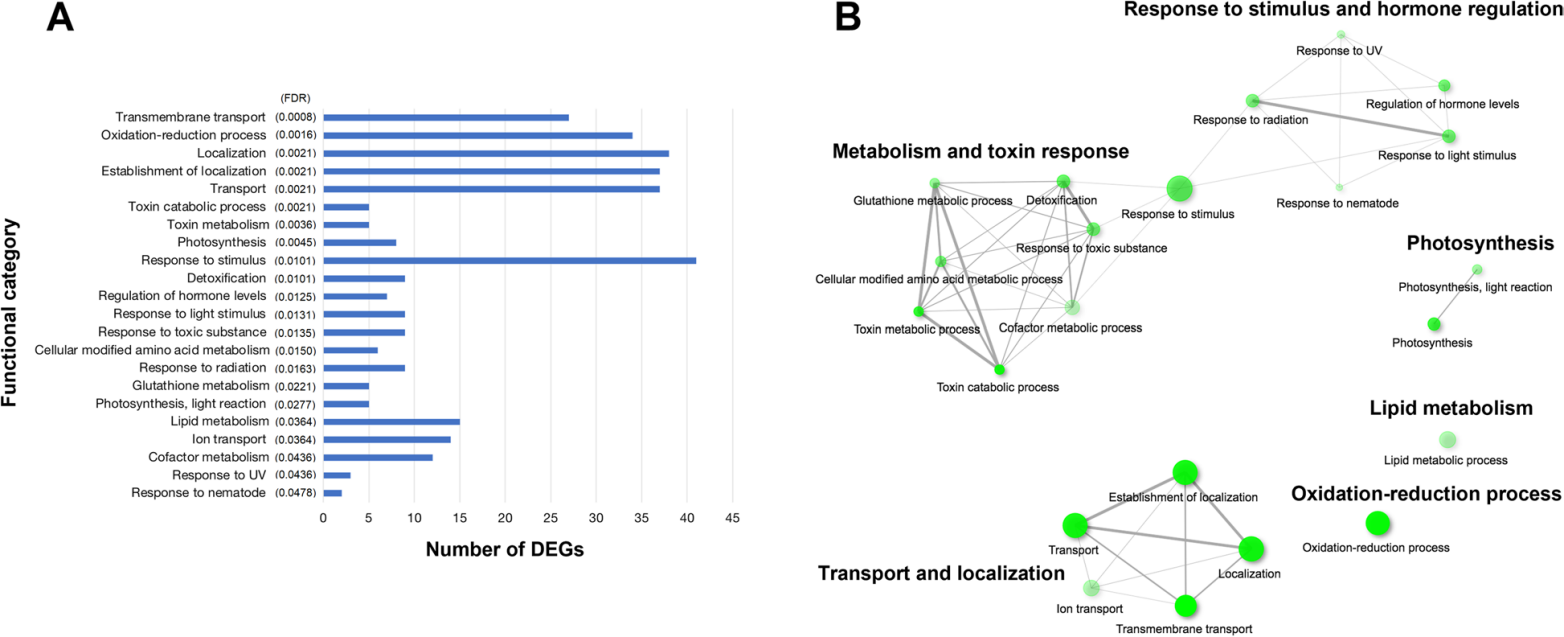
Gene Ontology (GO) enrichment analysis. **A** Significantly (FDR < 0.05) overrepresented GO terms-biological process for genes co-localizing with sorghum leaf angle QTL in the five genotypes (284 DEGs). **B** Relationships between enriched biological pathways. Green color intensity of nodes (circles) indicates significance level

Across the ten sorghum chromosomes, 69 QTL have been previously reported (Supplementary Table S2) from five studies that examined the genetic control of leaf angle for specific leaves [4, 5, 8–10] while Mantilla-Perez et al. [11] characterized the entire canopy. A total of 5146 unique genes were localized within the 69 QTL based on the physical coordinates (reference genome v3). A subset of 284 were DE for at least one layer comparison across all five sorghum genotypes, with chromosome 3 harboring the largest number (99), and chromosome 8 the least (four) (Supplementary Fig. S1, Supplementary Table S2, S3). For this set of 284 DE candidate genes, GO enrichment analysis identified 22 significantly (FDR < 0.05) overrepresented biological processes, with the top ten being transmembrane transport, oxidation-reduction process, localization, establishment of localization, toxin catabolism, toxin metabolism, photosynthesis, response to stimulus, detoxification, and regulation of hormone levels (Fig. 3A, Supplementary Table S4). The six major functional categories from the GO network graphs were transport and localization (38 genes), metabolism and toxin response (44 genes), response to stimuli and hormone regulation (42 genes), oxidation-reduction process (34 genes), lipid metabolism (15 genes), and photosynthesis (8 genes) with transport-related biological processes being the most significant (Fig. 3B). Visualization of gene expression patterns for each major functional category across genotype-canopy layer combinations using heatmaps revealed distinct clusters of genes with higher or lower expression in the top canopy compared to a layer below (Supplementary Fig. S2 A-F). Additionally, differences in gene expression magnitude were observed, with the UvsL layer consistently having the highest log2 fold change values for all genotypes and functional categories (Supplementary Fig. S2 A-F). Photosynthesis-related transcripts had overall lower expression levels in the top canopy relative to lower layers in all genotypes except PI656015 (MvsL and UvsL comparisons) (Supplementary Fig. S2C).

### Differentially expressed genes in the chromosome 7 QTL region containing Dw3

**Fig. 4:**
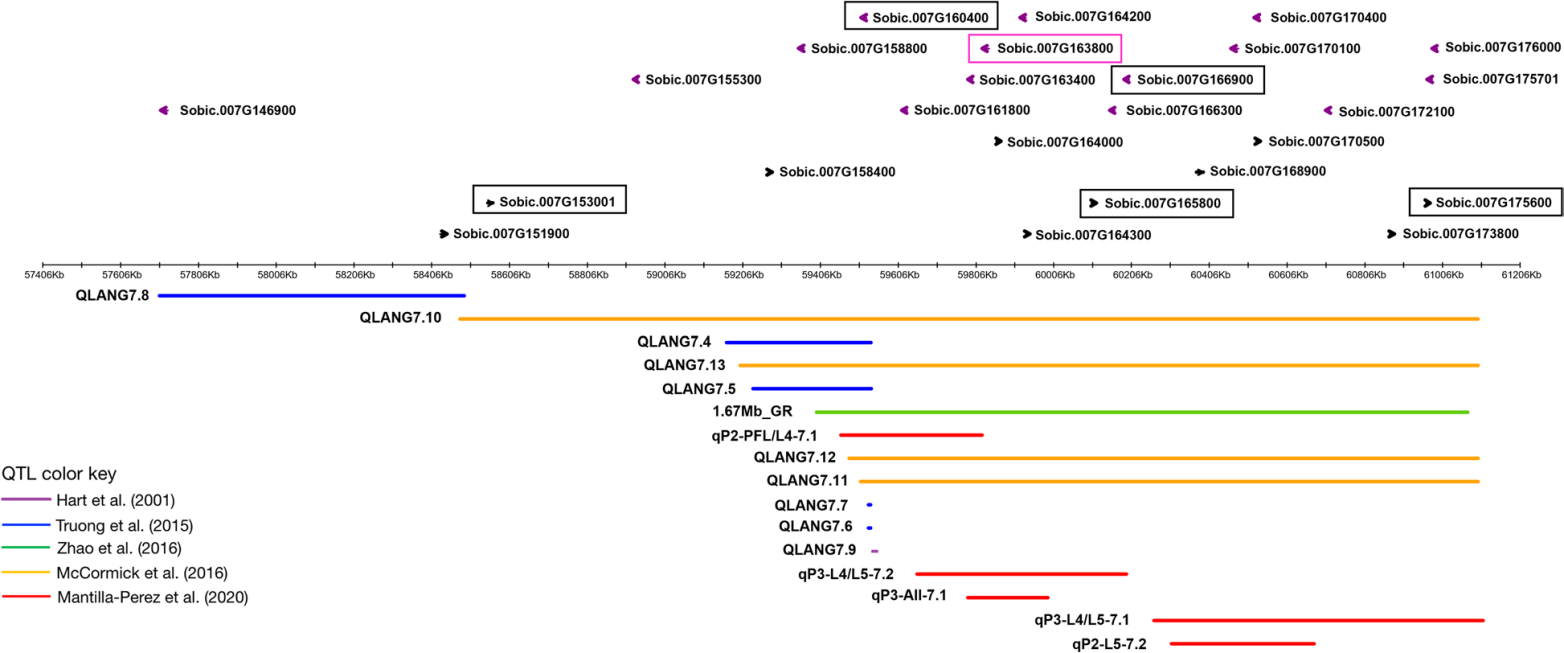
Top panel: Subset of 25 DEGs co-localizing with 16 QTL in the 3404.7 Kb chromosome 7 region containing the *Dw3* auxin efflux transporter (pink box). Black boxes indicate five candidates related to leaf angle. Lower panel: Color-coded leaf angle QTL. Detailed gene information is included in Table 1, Supplemental Tables S5 and S6

**Fig. 5:**
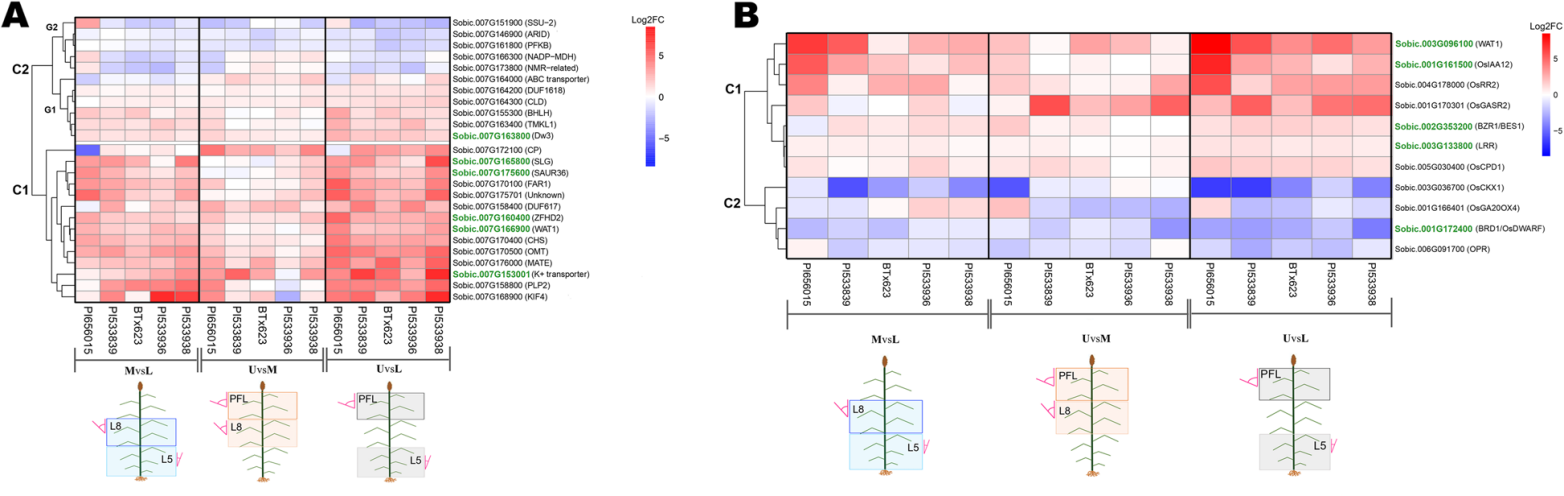
Heatmaps of DEGs co-localizing with known leaf angle QTL. **A** Expression patterns of 25 DEGs co-localizing with 16 QTL in the 3404.7 kb chromosome 7 region containing the *Dw3* auxin transporter. **B** Expression patterns of eleven hormonal-responsive candidate genes co-localizing with leaf angle QTL on other chromosomes. C1 and C2 indicate clusters 1 and 2, respectively. G1 and G2 indicate groups 1 and 2, respectively. MvsL-middle vs. lower layer comparison, UvsM-upper vs. middle layer comparison, and UvsL-upper vs. lower layer comparison. Homologs are indicated in parenthesis. Green font highlights novel candidate genes for layer-specific leaf angle manipulation presented in Fig. 6

Previous studies proposed that the *Dw3*-containing major-effect QTL on chromosome 7 (57706101–61,110,866) has additional genes contributing to leaf angle variation [5]. Therefore, this region with 16 overlapping QTL spanning 3404.7 Kb was further investigated (Fig. 4, Supplementary Fig. S1), and 24 genes DE between canopy layers identified, in addition to *Dw3* (Figs. 4 and 5A, Supplementary Fig. S1). Analysis of expression patterns revealed two distinctive clusters. Cluster 1 contained 14 genes with overall higher expression in the top canopy relative to the layers below, with the highest expression magnitudes observed for the UvsL comparison (Fig. 5A). The remaining 11 genes constituted cluster 2, with varying expression profiles between two distinct groups. Group 1 included six genes with higher expression in the top canopy compared to lower layers, but overall lower magnitudes than cluster 1. The other five genes in cluster 2-group 2 had overall lower expression than cluster 2-group 1 for all layer comparisons and genotypes (Fig. 5A). Following the prioritization criteria described in Methods, a subset of five genes in this QTL region, besides *Dw3*, were selected as the most promising candidates, which are involved in transport, hormone response (auxin and BR), or act as transcription factors (Table 1; Supplementary Table S5, Supplementary Table S6).

**Table 1:** Novel candidate genes co-localizing with QTL in the *Dw3* region on chromosome 7

Sorghum gene id	Sorghum gene description	Layer comparison	Co-localizing QTL^**a**^	Rice homolog	Rice homolog description
**Candidates downstream of** ***Dw3***					
*Sobic.007G165800*	BAHD acyltransferase DCR	MvsL UvsL	*QLANG7.10 QLANG7.11 QLANG7.12 QLANG7.13* *1.67Mb_GR* *qP3-L4/L5–7.2*	*Os08g0562500* (E value: 0.0, Identity: 84.7%)	- BAHD acyltransferase-like protein. - *Slender Grain* (*SLG*). - Control of grain size and leaf angle. - Regulation of BR homeostasis.
*Sobic.007G166900*	*WALLS ARE THIN 1* (*WAT1*) EamA-like transporter family	MvsL UvsL	*QLANG7.10 QLANG7.11 QLANG7.12 QLANG7.13* *1.67Mb_GR* *qP3-L4/L5–7.2*	*Os08g0561500* (E value: 7e-141, Identity: 84.0%)	- *WALLS ARE THIN 1* (*WAT1*) - Similar to nodulin-like protein 5NG4 - Usually Multiple Acids Move In and out Transporter 12*: OsUMAMIT12*)
*Sobic.007G175600*	Small auxin up-regulated RNA (*SAUR*) 36.	MvsL UvsL	*QLANG7.10 QLANG7.11 QLANG7.12 QLANG7.13* *1.67Mb_GR* *qP3-L4/L5–7.1*	*Os08g0550700* (E value: 9e-64, Identity: 71.4%)	Similar to Auxin induced protein (*OsSAUR36*)
**Candidates upstream of** ***Dw3***					
*Sobic.007G153001*	Potassium ion (K+) transporter	UvsM MvsL UvsL	*QLANG7.10*	*Os08g0466200* (E value: 0.0, Identity: 86.1%)	Similar to KUP-related potassium transporter (*OsHAK4*)
*Sobic.007G160400*	Zinc-finger homeodomain protein 2	UvsL	*QLANG7.4 QLANG7.5 QLANG7.10 QLANG7.11 QLANG7.12 QLANG7.13* *1.67Mb_GR* *qP2-PFL/L4–7.1*	*Os08g0479400* (LOC_Os08g37400) (*OsZHD2*). (E value: 6e-174, Identity: 83.4%)	Similar to Hydroxyproline-rich glycoprotein DZ-HRGP precursor. Zinc-finger homeodomain protein 2-like (LOC4345845), mRNA)

Only rice homologs are shown. Supplementary Tables S5 and S6 show the complete list of genes co-localizing with QTL, and maize and Arabidopsis homologs. ^a^QTL studies include [4, 5, 8–11] (Supplementary Table S2)

### Hormonal-responsive DEGs co-localize with QTL on other chromosomes

Considering the known hormonal regulation of leaf angle, phytohormone-related DEGs co-localizing with leaf angle QTL were examined. Eleven genes were identified with this predicted general function within 12 QTL on chromosomes 1 through 6 (Fig. 5B, Supplementary Table S7). Cluster 1 includes seven genes with higher expression in the top canopy relative to lower layers: (i) the homologs of auxin responsive genes *Sobic.001G161500* (Aux/IAA transcription factor-*IAA12*) and *Sobic.003G096100* (*WALLS ARE THIN 1*-*WAT1*); (ii) the predicted negative regulator of cytokinin signaling *Sobic.004G178000*, homologous to *OsRR2*; (iii) *Sobic.001G170301*, homologous to the GA-stimulated transcript *OsGASR2*; and (iv) three predicted BR biosynthesis and signaling genes: *Sobic.005G030400*-cytochrome P450 90A1 and homologous to *OsCPD1*, *OsCPD2*, and Arabidopsis *DWF3*, *Sobic.003G133800* predicted to encode a leucine-rich repeat protein associated with BRASSINOSTEROID INSENSITIVE 1-ASSOCIATED RECEPTOR KINASE 1 (*BAK1*) and *Sobic.002G353200,* the well-studied *BZR1/BES1* transcription factor (Fig. 5B and Supplementary Table S7). Cluster 2 comprised of four genes with consistently lower expression in the top layers than in the lower canopy, including: (i) *Sobic.001G172400*, a cytochrome P450 85A1 homologous to the rice *brassinosteroid-deficient dwarf1* (*brd1/CYP85A1*) gene and maize BR C-6 oxidase (*BRD1*) that catalyzes the C6 oxidation step during BR biosynthesis; (ii) *Sobic.003G036700,* a homolog of *OsCKX1,* which encodes a cytokinin dehydrogenase 1, an enzyme that inactivates cytokinin by irreversible degradation; (iii) *Sobic.001G166401* with homology to a rice *gibberellin 20 oxidase 4 (Os03g0618300)*, which catalyzes oxidation steps during GA biosynthesis, and (iv) *Sobic.006G091700*, proposed to encode a putative 12-oxophytodienoate reductase involved in JA biosynthesis (Fig. 5B, Supplementary Table S7).

## Discussion

The observed leaf angle distribution in sorghum is opposite to the proposed “smart canopy” ideotype, which reveals the need for improving this trait to increase grain and biomass yield. We conducted a transcriptional profiling of genes across canopy layers using collar tissue, whose cells ultimately determine leaf angle (Supplementary Fig. S5). The list of DEGs between canopy layers was refined based on their co-localization with previously known leaf angle QTL to propose candidates for future validation and manipulation to develop sorghums with superior canopy architecture. We have confirmed that the known role of *Dw3* in leaf angle determination across the canopy [11] is also significant at the transcriptome level, and the observed increasing expression from lower to upper layers is consistent with previous results obtained by RT-qPCR [11]. Multiple pieces of evidence substantiate the importance of dissecting the 3404.7 Kb chromosome 7 region containing *Dw3* for the efficient manipulation of leaf angle: (i) the abundance of overlapping QTL with major effects on the trait (Fig. 4); (ii) the previously reported marker-trait associations on this interval that did not include *Dw3* [5]; and (iii) the identification of QTL from a biparental population that did not segregate for *Dw3* [4]. Therefore, focusing the transcriptome analysis on this region, we identified 24 DEGs across layers out of the total 321 genes localized in the 57,706,101–61,110,866 bp interval, with nine genes upstream and 15 downstream of *Dw3* (Fig. 4). Additionally, from the 259 DEGs identified in other chromosomes (Supplementary Fig. S1), we have highlighted eleven hormonal-responsive genes co-localizing with QTL on chromosomes 1 through 6 (Fig. 5B). Based on homology with genes known to control leaf angle or cell growth and expression-phenotype associations, eleven candidates are emphasized and described below.

Even though sorghum homologs of liguleless genes were not co-localized with previously reported leaf angle QTL, their expression profiles were investigated because of their known association with our target trait in cereals [2]. Differential expression analysis of *Sobic.006G247700* (*LG1*), *Sobic.003G363600* (*LG2*), *Sobic.003G144200* (*LG3*), *Sobic.009G030200* (*LG4*) and *Sobic.010G137400* (*LGN-R*) across canopy layers revealed that *LG1* and *LG2* were DE for the UvsM and UvsL comparisons for almost all genotypes (Supplementary Fig. S3).

### Candidates based on homology with genes known to affect leaf angle in related species

#### Upper and middle layers

**Fig. 6:**
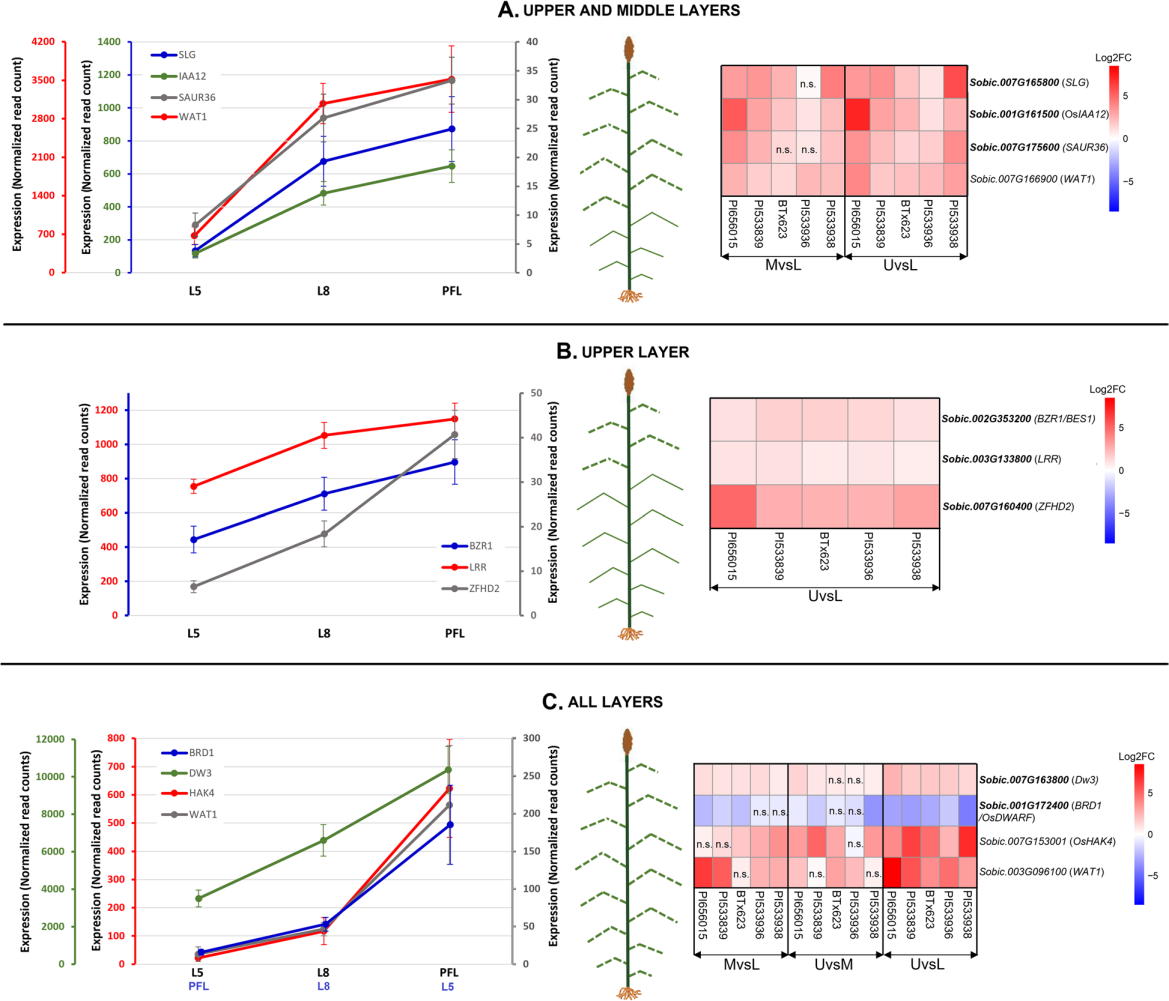
Top 11 candidate genes proposed for canopy layer-specific leaf angle improvement. **A** Upper and middle layers (four genes). **B** Upper layer (three genes). **C** All layers (four genes). Bolded genes or homologs are known/proposed to control leaf angle in sorghum or other species. Non-bolded genes are involved in biological processes related to cell size/expansion, shape and/or cell number. All proposed candidate genes are novel in sorghum except *Dw3* and *BZR1/BES1*. Heatmaps show log2 fold change of expression between canopy layers. Graphs show similar expression patterns across layers of candidates summarized as average normalized read counts over genotypes. Axis color in graphs corresponds to gene color. n.s.: non-significant

The two strongest candidates to be exploited to alter leaf angle in these two layers are *Sobic.007G165800* (*SLG* homolog) and *Sobic.001G161500* (*OsIAA12* homolog). Both genes were highly and significantly expressed in the upper and middle canopy relative to the lower layer (UvsL and MvsL comparisons) and co-localized with eight QTL on chromosomes 7 and 1, respectively (Fig. 5A and B, Table 1, Supplementary Table S7). In rice, *SLG* controls leaf angle and grain size by regulating brassinosteroid homeostasis, as demonstrated by SLG gain-of-function mutants which had increased leaf angles and adaxial collar cell lengths [21]. Similarly, overexpression of *OsIAA12* in rice increased leaf angle, and analysis of longitudinal collar sections revealed the presence of larger adaxial parenchyma cells [22]. These expression-angle relationships reported in rice agree with those observed in sorghum for *Sobic.007G165800* and *Sobic.001G161500* across layers (Fig. 5A and Fig. 6A), making them strong candidates for leaf angle improvement of the upper and middle layers.

#### Upper canopy

Two BR-related genes (*Sobic.002G353200*-*BZR1/BES1* and *Sobic.003G133800)* are the strongest candidates to manipulate angle of the upper canopy by altering their naturally higher expression relative to the lower layer (UvsL comparison) (Fig. 6B). In sorghum, *BZR1/BES1* has been previously associated with variation in angle of the pre-flag leaf [10], and increasing expression from lower to upper canopy layers has been reported based on RT-qPCR [11]. This positive trend between leaf angle and expression across layers are consistent with observations in other species. E.g., silencing the *BZR1/BES1* rice homolog (*Os07g0580500*, *OsBZR1*) by RNAi resulted in more erect leaves and plants with reduced sensitivity to external BR applications [23]. *Sobic.003G133800,* an *LRR* predicted as a BAK1-like gene, is a strong candidate for canopy improvement because downregulating the expression of this BR signaling gene in rice caused plants with erect leaves and normal seed production [24]. Their co-localization with two overlapping QTL, trends of higher expression in the upper canopy with larger angles consistent with reports in other species (Fig. 6B), and the previously reported association of BR genes with leaf angle variation in sorghum [10, 11] make *Sobic.002G353200* and *Sobic.003G133800* interesting targets for functional validation and layer-specific silencing/downregulation to decrease leaf angle in the upper canopy.

#### Throughout the canopy

Two genes known to control leaf angle in sorghum and other species were DE in all layer comparisons (MvsL, UvsM, and UvsL), and thus, represent interesting targets to reverse the overall distribution of leaf angle across the canopy, to achieve the proposed ideotype. *Dw3* is an auxin efflux transporter with the nonfunctional allele (*dw3/dw3*) causing overall smaller angles than thefunctional allele (*Dw3/Dw3*) [4, 8, 13]. In our study, the higher *Dw3* expression observed in the upper and middle layers are consistent across lines irrespective of the function/non-functional allele (Fig. 6C). Therefore, targeted manipulation of *Dw3* expression in collars by utilizing techniques such as the CRISPR-based tissue-specific knockout system [25] could alter auxin transport and growth only in collars contributing to the development of genotypes with the proposed leaf angle ideotype without having pleiotropic effects on plant height. The second candidate, *Sobic.001G172400* (*BRD1* homolog)*,* had lower expression in the upper and middle canopy compared to lower layers (Fig. 6C) and thus, a negative association between expression and phenotype for all genotypes. This result is not in agreement with observations in other species. In rice, *brd1* mutants are defective in BR biosynthesis, have erect leaves, and other developmental defects such as shorter internodes, reduced plant height, and altered cell/plant morphology [26]. Introduction of the functional C-6 oxidase, *OsDWARF*, into *brd1* mutants partially restored normal growth [26]. In maize, *brd1* mutants exhibited developmental abnormalities resulting from inhibition of cell elongation, and phenotypes were rescued by brassinolide application [27]. In spite of the opposite expression-phenotype association across layers in sorghum, further investigation of *Sobic.001G172400* and its role in leaf angle determination would be desirable based on the other pieces of evidence revealed herein about this gene.

### Candidates based on homology with genes affecting biological processes related to leaf angle

Because leaf angle is determined by the size of collar cells, manipulation of genes affecting cell size/expansion, shape, and/or cell number are promising targets to achieve the proposed ideotype.

#### Upper and middle layers

Two auxin-responsive genes located downstream of *Dw3* (Fig. 4), *Sobic.007G175600* and *Sobic.007G166900*, have higher expression in the middle and upper canopies compared to the lower layer (Table 1, Fig. 5A, Fig. 6A). *Sobic.007G175600* (*SAUR36* homolog) belongs to a 71-member family in sorghum [28] and has previously been proposed as a candidate controlling angle of the PFL [5]. In Arabidopsis, *SAURs* inhibit PP2C-D phosphatases to activate plasma membrane H+ ATPases, promoting cell expansion during acid growth [29–31]. Overexpression of the Arabidopsis *SAUR36* increased cell expansion, and thus, hypocotyl cell lengths [32]. Interestingly, *saur36* T-DNA loss of function Arabidopsis mutants had larger leaves [33], suggesting that *SAUR36* might be self-regulating and the effects on cell elongation might also be tissue-dependent [29]. Previous studies have also reported that SAUR proteins are regulated by phytohormones and are involved in the auxin-BR crosstalk [29]. In Arabidopsis, *BES1* binds to *SAUR36* [34], suggesting it could be a downstream effector that mediates, at least in part, BR-related cell expansion [29]. In our study, a negative co-expression relationship was identified between *SAUR36* and *Sobic.009G040700*, predicted as a *BRI1 suppressor 1* (*BSU1*)*-*like gene (Supplementary Fig. S4) and regulator of BR signaling. In Arabidopsis, *BSU1* dephosphorylates BR INSENSITIVE 2, an inhibitor of the BR signaling pathway [35, 36], causing its inactivation and leading to the accumulation of unphosphorylated *BES1/BZR1*, and enhanced BR signaling [37]. This body of evidence makes *SAUR36* a strong candidate gene, potentially affecting the expansion of collar cells and thus, increasing leaf angle. Therefore, targeted knockdown of the sorghum *SAUR36* in collar tissues to reduce cell size and number while minimizing effects on other tissues might reduce leaf angle for the middle and upper layers, resulting in the more desirable ideotype.

The second candidate gene*, Sobic.007G166900*, is homologous to Arabidopsis *Walls Are Thin 1* (*WAT1*-*AT1G75500)*, a vacuolar auxin transporter involved in auxin homeostasis and secondary cell wall biogenesis [38, 39]. The Arabidopsis *WAT1* is co-expressed with several auxin-related genes, and its mutation caused a defect in cell elongation (dwarfism) and smaller leaf surfaces [38, 39]. In our study, *Sobic.007G166900* had higher expression in the top canopy compared to the lower layer, and its downregulation might result in the size reduction of collar cells, with the potential to alter leaf angle in the top canopy. In summary, the angle-expression trend observed across layers and consistent with those reported in other species, the co-localization with leaf angle QTL (Fig. 6A, Supplementary Fig. S1) and homology with cell size-related genes make *Sobic.007G175600* and *Sobic.007G166900* strong candidates for leaf angle modification (Fig. 6A).

#### Upper canopy

*Sobic.007G160400*, a zinc finger homeodomain 2 (*ZFHD2*) transcription factor, has higher expression in the upper canopy compared to the lower layer (Fig. 6B), and it was previously proposed to control the angle of the PFL [5]. In rice, overexpression of the *Sobic.007G160400* homolog *OsZHD2*, or *OsZHD1*, a paralog of *OsZHD2* with over 80% protein sequence identity, caused abaxial leaf curling [40]. This phenotype change was attributed to the increased numbers of abaxial bulliform cells in an altered arrangement compared to the wild type [40]. Therefore, the changes in plant architecture associated with the manipulation of its rice homologs and the consistent trend of leaf angle-expression observed for all genotypes (Fig. 6B) make *Sobic.007G160400* a strong candidate for leaf angle modification in the upper canopy.

#### Throughout the canopy

*Sobic.007G153001* and *Sobic.003G096100* have higher expression in upper canopies relative to lower layers (Fig. 5A, B, and Fig. 6C) and could offer the opportunity to reverse the undesirable distribution of leaf angle by inverting their expression profiles across the canopy and achieve the proposed ideotype. *Sobic.007G153001* is located upstream of *Dw3* and homologous to *OsHAK4,* a potassium (K+) transporter (Fig. 4, Table 1). The *KT/KUP/HAK* (*High-Affinity K+ Transporter*) family of potassium transporters has multiple roles, including regulation of plant growth and development [41]. Potassium ions facilitate plant growth by providing the necessary osmotic potential for cellular water uptake, increasing turgor pressure, and driving cell expansion [18, 19]. Using maize coleoptiles, Claussen et al. [18] demonstrated that K+ ions are required for auxin-induced growth and blocking K+ obliterated growth. Although there is no previous evidence relating *OsHAK4* to leaf angle, changes in other plant architecture traits resulting from modification of *HAK* potassium transporters have been reported. For instance, the inactivation of *OsHAK5* in rice decreased polar auxin transport in shoots and roots, reduced tiller numbers and root length [42]. Additionally, EMS mutation of *KT2/KUP2/SHY3* in Arabidopsis reduced cell expansion causing short hypocotyls, small leaves, and short flowering stems [43]. Therefore, based on the transcriptome evidence obtained in this study and the known roles of K+ transporters in other species, validating the effect of *Sobic.007G153001* in leaf angle determination could open a novel path to alter this trait across the canopy. The second candidate in this group, *Sobic.003G096100* (*WALLS ARE THIN 1*)*,* is homologous to *AT3G18200*, a nodulin MtN21/EamA-like transporter family protein (*UMAMIT4*) involved in transmembrane transport, that interacts with transcription factors involved in secondary cell wall synthesis [44]. The consistent higher expression of *Sobic.003G096100* in the upper canopy compared to lower layers (Fig. 6C), co-localization with two QTL, and gene function make it a strong candidate for leaf angle improvement.

## Conclusions

We have conducted a transcriptome analysis to identify genes controlling leaf angle at specific canopy layers and generated new insights on the expression profiles of promising candidates that can be leveraged to achieve the “smart canopy” ideotype in sorghum. The identification of 284 DEGs between canopy layers that co-localized with known QTL demonstrates the intricate genetic architecture of leaf angle and the need to avoid the oversimplification of characterizing a single leaf to represent the entire canopy. Based on predicted gene function, homology, phenotype-expression trends across layers, and consistency in expression across genotypes, the most promising candidates for layer-specific leaf angle improvement were further narrowed down to eleven genes. Seven of these genes are known to affect leaf angle in other species, while the other four are involved in biological processes related to leaf angle changes such as cell size/expansion, shape, and cell number. Considering that the manipulation of some candidates could have undesirable pleiotropic effects, the characterization of their effects in other tissues across developmental stages should precede their exploitation to alter leaf angle according to the proposed ideotype. Alternatively, genetic engineering or editing strategies designed to induce expression changes specifically in collar cells could be instrumental to minimize alterations in other traits and successfully realize a “smart canopy” crop.

## Methods

### Plant material, experimental design, and phenotyping

Five sorghum genotypes (PI656015, PI533839, BTx623, PI533936, and PI533938) from the sorghum association panel [45] were selected for this study, based on their alternative haplotypes for the chromosome 7 region that contains the *Dw3* gene and overall differences in leaf angle: (1) PI656015 and PI533839 with functional haplotype-*Dw3*/*Dw3* and large leaf angle, and (2) BTx623, PI533936, and PI533938 with nonfunctional haplotype-*dw3*/*dw3* and small leaf angle [10, 11]. Experiments were set up in a completely randomized design with six plants per genotype and conducted in a greenhouse at 28 °C–30 °C and 13 h light:11 h dark photoperiod. A total of 30 6-L pots were filled with 2.4 kg of MetroMix 900 pot-mix (SunGro Horticulture), and plants were fertilized once a week by adding Peters® Excel Cal-Mag Fertilizer (15–5-5) to the irrigation water. Leaf angle was measured for all fully expanded leaves as the inclination between the midrib of the leaf blade and the stem using a barcoded paper protractor, a barcode scanner, and a tablet during plant development. Leaf angle data were collected weekly until anthesis (average 67 days after planting).

### Collar tissue collection

Collar tissues were collected from leaf 5 (L5), representing the lower canopy, leaf 8 (L8) representing the middle canopy, and the pre-flag leaf (PFL) representing the upper canopy during plant development as soon as the respective leaves were fully expanded. For each genotype-canopy layer combination, two collars of the respective leaf from two plants were pooled for each of the three biological replicates, placed in a 5 mL sterile Eppendorf® tube, flash-frozen in liquid nitrogen, and stored in a − 80 °C freezer. To grind frozen collar tissue, two sterile 5/32″ diameter stainless-steel balls were placed in the Eppendorf® tube with tissue and ground using a GenoGrinder® 2010 set at 700 rpm for 30 s. During grinding, samples were periodically placed in liquid nitrogen to avoid thawing, ground repeatedly (approximately three times per sample) until the tissue was completely ground and stored in a − 80 °C freezer. In total, 45 samples were collected for RNA-seq (five sorghum genotypes x three replications x three canopy layers).

### RNA preparation and RNA-seq

RNA was extracted from the ground collar tissue samples described above using the Qiagen® RNeasy® Plant Mini Kit (QIAGEN, Valencia, CA). Extracted RNA was DNAse treated using the Ambion® TURBO DNA-free kit™ (Invitrogen, Carlsbad, CA) to remove genomic DNA contamination. RNA clean-up was done for all DNAse-treated RNA samples using the Qiagen® RNeasy® MiniElute Cleanup Kit. RNA concentration was quantified using a NanoDrop spectrophotometer 2000 (NanoDrop Technologies, Wilmington, DE). RNA integrity was checked using the Agilent® 2100 Bioanalyzer™, and all samples were of good quality with an RNA integrity of 8 or greater. RNA samples were submitted to the DNA facility at Iowa State University for multiplex library preparation and single-end sequencing using the Illumina HiSeq 3000 instrument at a read length of 150 base pairs.

### Bioinformatics and statistical analysis

FastQC (http://www.bioinformatics.babraham.ac.uk/projects/fastqc/) was used to assess the quality of reads. For each Fastq data file, Trimmomatic, v0.36 [46] was used to trim the 150 base pair reads to remove adaptors, bases of quality score < 33, and reads shorter than 30 bp using the following steps: ILLUMINACLIP (fasta = TruSeq2-SE.fa; SeedMismatches = 2; PalindroneClipThreshold = 30; SimpleClipThreshold = 10); LEADING = 10; TRAILING = 10; MAXINFO (TargetLength = 36; Strictness = 0.5); MINLEN = 36. Before sequence alignment, the BTx623 v3 reference genome files (Sorghum_bicolor.Sorghum_bicolor_NCBIv3.dna.toplevel.fa and Sorghum_bicolor.Sorghum_bicolor_NCBIv3.46.gff3.gz) [20] were downloaded from Ensembl Plants (http://plants.ensembl.org), and Cufflinks v2.2.1 used to convert the reference genome file from gff3 to gtf format. RNA-Seq by Expectation Maximization (RSEM v1.3) [47] and Bowtie 2 [48] were utilized to prepare the reference genome, align the processed single-end reads to the reference genome, and estimate expression levels under the default settings. Supplementary Fig. S1 shows a summary of our RNA-seq data analysis pipeline. Differential expression analyses for canopy layer comparisons were done separately for each sorghum genotype using the statistical program R [49]. The Bioconductor package EdgeR [50, 51] was used for data normalization across all canopy layers for each sorghum genotype using the Trimmed Mean of M (TMM) values [52]. EdgeR was utilized for single factor, pairwise canopy layer comparisons for each sorghum genotype to determine differential gene expression at false discovery rate (FDR) < 0.05. The comparisons are referred herein as MvsL (middle vs. lower), UvsM (upper vs. middle), and UvsL (upper vs. lower). The graphics package, ggplot2 [53], facilitated the comparison of replicates for each genotype and canopy layer combination to ensure replicability. Using ggplot2, graphs such as principal component and biological coefficient of variation were generated to visualize similarity among replicates. Differences in gene expression were quantified as the logarithm of the ratio of mean normalized counts between canopy layers (log fold change). The overlap among differentially expressed (DE) gene sets was determined by Venn diagrams drawn using jVenn (http://jvenn.toulouse.inra.fr/app/index.html) [54].

### Analysis of DE genes (DEGs) co-localizing with leaf angle QTL

DEGs co-localizing with known leaf angle QTL were identified for each canopy layer comparison. Sorghum genome v3 QTL physical coordinates and genes under QTL for four sorghum leaf angle studies [4, 8–10] were obtained from the Sorghum QTL Atlas [55]. For Mantilla-Perez et al. [11] and Zhao et al. [5], physical QTL coordinates were first converted from v1 to v3 using the Ensembl Plants assembly converter tool (https://plants.ensembl.org/index.html), and genes under each QTL were extracted using the BioMart tool.

ShinyGO [56] was used to conduct gene ontology (GO) enrichment analysis for candidate genes co-localizing with known leaf angle QTL, identifying significantly overrepresented biological processes (FDR < 0.05). Enrichment analysis was based on hypergeometric distribution followed by FDR correction. Significant GO terms (biological process) were used to generate GO network graphs and visualize the relationships between enriched pathways. Connected nodes with related biological processes sharing 20% or more genes were identified, and gene sets grouped to remove duplicates. Heatmaps, clustered by row (similarity in gene expression) were constructed for each functional category using the pheatmap package version 1.0.12 [57] in R [49] to visualize gene expression patterns for respective canopy layer comparisons across the five sorghum genotypes (15 comparisons total). Gene annotations were obtained from The National Center for Biotechnology Information-NCBI (https://www.ncbi.nlm.nih.gov/), Phytozome.net (https://phytozome.jgi.doe.gov/pz/portal.html), and Ensembl Plants (https://plants.ensembl.org/index.html). Additionally, rice, maize, and Arabidopsis homologs of sorghum genes were identified using gProfiler [58] and The Arabidopsis Information Resource (TAIR: https://www.arabidopsis.org/). The Basic Local Alignment Search Tool (BLAST and tBLASTn) in NCBI was used to validate candidate gene sequence homology between sorghum and other species. The sorghum functional genomics database [59] was used to check for interactions between identified candidate genes and other leaf angle-related genes.

### Identification of candidate genes

**Fig. 7:**
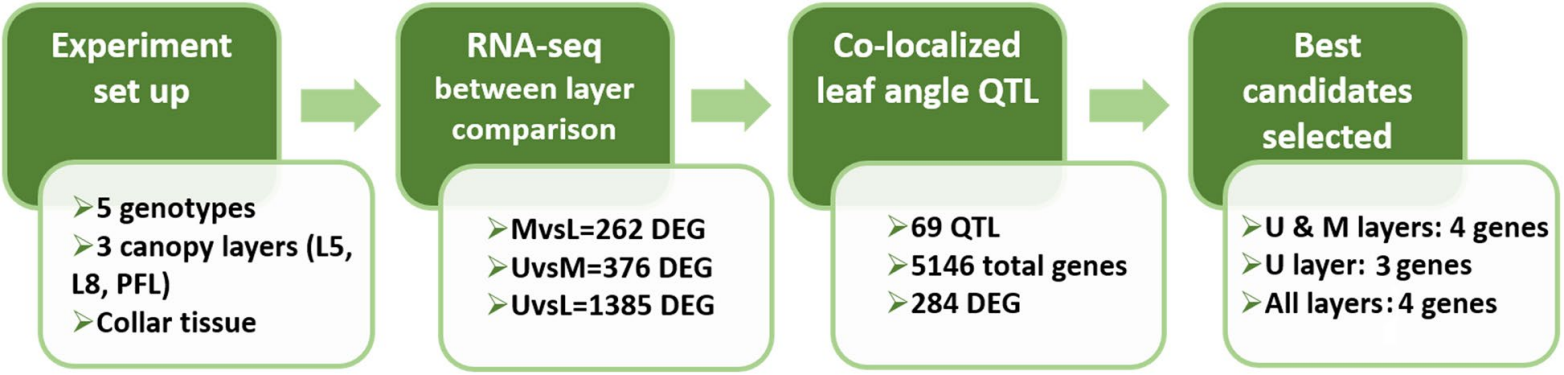
Overview of experimental setup, data analysis pipeline, and selection criteria to identify the top candidate genes for canopy layer-specific leaf angle improvement. DEG: differentially expressed gene. U: upper canopy layer. M: middle canopy layer. L: lower canopy layer

The most promising candidate genes for future validation and the development of an improved canopy architecture were selected based on the following criteria: (a) co-localization with previously reported leaf angle QTL, (b) differential expression in at least three genotypes for each layer comparison, (c) function related to leaf angle control in sorghum or other species (homology to rice, maize, and Arabidopsis), (d) homology to genes with biological functions related to leaf angle, i.e., cell size/expansion, shape, and cell number, and (e) expression levels across layers in agreement with expression-leaf angle associations reported for homologs in related species. Finally, sets of candidate genes to alter leaf angle are proposed for: (a) the upper layer, based on significance for the UvsL comparison, (b) both the upper and middle layers, based on the MvsL and UvsL comparisons, and (c) all layers, based on their DE in all pairwise comparisons, MvsL, UvsM, and UvsL. Figure 7 shows an overview of the experimental setup, data analysis pipeline, and selection criteria for the top candidate genes for canopy layer-specific leaf angle improvement.

## Supplementary Information

**Additional file 1: Supplementary Figure S1.** Differentially expressed genes co-localizing with leaf angle QTL on each chromosome.

**Additional file 2: Supplementary Figure S2.** A-F. Expression patterns for DEGs co-localizing with leaf angle QTL (grouped by functional category).

**Additional file 3: Supplementary Figure S3**. Heatmap summarizing expression patterns of genes of the liguleless family *Sobic.006G247700* (*LG1*), *Sobic.003G363600* (*LG2*), *Sobic.003G144200* (*LG3*), *Sobic.009G030200* (*LG4*) and *Sobic.010G137400* (*LGN-R*).

**Additional file 4: Supplementary Figure S4.** *Sobic.007G175600* (*SAUR36*) and interacting genes. Source: Sorghum FDB (Tian et al., 2016). *SAUR36* interacts with *Sobic.009G040700*-BRI1 suppressor 1 (*BSU1*)-like 1 marked with an asterisk.

**Additional file 5: Supplementary Figure S5.** Collar tissue from sorghum plant as an illustration of the tissue used for RNA extraction.

**Additional file 6: Supplementary Figure S6.** RNA-seq pipeline used for the study.

**Additional file 7: Supplementary Table S1.** RNA-seq data summary.

**Additional file 8: Supplementary Table S2.** Sorghum leaf angle QTL (69), number of DEGs co-localizing with QTL on each chromosome, and references.

**Additional file 9: Supplementary Table S3.** Differentially expressed genes co-localizing with leaf angle QTL (shown in Supplementary Fig. S2 graphs).

**Additional file 10: Supplementary Table S4.** GO terms (biological process) for the 284 DEGs co-localizing with leaf angle QTL.

**Additional file 11: Supplementary Table S5.** Annotations for the 284 candidate DEGs co-localizing with leaf angle QTL (genes are grouped by chromosome).

**Additional file 12: Supplementary Table S6.** Rice, maize, and Arabidopsis homologs for the 284 DE candidate genes co-localizing with leaf angle QTL. Note: A separate excel file with these Tables is provided.

**Additional file 13: Supplementary Table S7.** Hormonal-responsive candidate genes co-localizing with leaf angle QTL on other chromosomes, and homologs in rice (*Oryza sativa*-*Os*), maize (*Zea mays*-*Zm*), and Arabidopsis (*Arabidopsis thaliana*-*At*).

## Data Availability

Raw RNA-seq data files (Fastq files) for this study are available at the NCBI Sequence Read Archive database under the BioProject submission number PRJNA681303. Sorghum lines used in this project are publicly available from the National Genetic Resources Program at the United States Department of Agriculture (USDA/ARS).

## Abbreviations

QTL: Quantitative trait loci
BRs: Brassinosteroids
RNA-seq: RNA sequencing
FDR: False discovery rate
MvsL: Middle vs. lower canopy layer comparison
UvsM: Upper vs. middle canopy layer comparison
UvsL: Upper vs. lower canopy layer comparison
GO: Gene ontology
DE: Differentially expressed
DEGs: Differentially expressed genes
L5: Leaf 5
L8: Leaf 8
PFL: Pre-flag leaf
