# Supplementary material for: Dissection of canopy layer-specific genetic control of leaf angle in Sorghum bicolor by RNA sequencing
Source: BMC Genomics. 2022 Feb 3;23:95. doi: 10.1186/s12864-021-08251-4 (PMC8812014; doi:10.1186/s12864-021-08251-4)
Supplement: Supplementary file 1 — Additional file 1: Supplementary Figure S1. Differentially expressed genes co-localizing with leaf angle QTL on each chromosome. [file 12864_2021_8251_MOESM1_ESM.docx]

**Supplementary Fig.(s) S1.** Differentially expressed (DE) genes (FDR<0.05) co-localizing with leaf angle QTL on each chromosome.

General description for all graphs included in Supplementary Fig.(s) S1. Separate figures are drawn for each chromosome and in some instances, genomic regions for one chromosome are divided into multiple segments (regions 1, 2 and 3). Genes are represented above the chromosomal region using arrows (**>** represents forward direction and **<** represents reverse direction) and corresponding numbers are shown in Supplementary Table S3. Candidate genes proposed in the manuscript are indicated in boxes. Color-coded QTL reported by each study are indicated by lines below each chromosomal region ( Hart *et al*., 2001; Mantilla Perez *et al*., 2014; Truong *et al*., 2015; McCormick *et al*., 2016; Zhao *et al*., 2016; Mantilla-Perez *et al*., 2020).

**Chromosome 1:** 11 QTL total, 68 DEGs (1-68).

Chr1: Region 1 (genes 1-28).

*brd1* homolog

*OsIAA12* homolog

*OsGA20OX4* homolog

*OsGASR2* homolog

Chr1: Region 2 (genes 29-68).

**Chromosome 2:** 3 QTL; 7 DEGs (genes 69-75).

*BZR1/BES1*

**Chromosome 3:** 14 QTL, 99 genes (76-174).

Chr3: Region 1 (genes 76-127).

*OsUMAMIT1*/ *WAT1* homolog

*LRR* protein

*OsCKX1* homolog

Chr3: Region 2 (genes 128-160).

Chr3: Region 3 (genes 161-174).

**Chromosome 4:** 4 QTL, 28 DEGs (175-202).

*OsRR2* homolog

**Chromosome 5:** 3 QTL, 14 genes (203-216).

*OsCPD1/OsCPD2* homolog

**Chromosome 6:** 6 QTL, 22 DEGs (217-238).

*OPR*

**Chromosome 7:** 17 QTL total 30 genes (239-268).

Chr7: Region 1 (genes 239-243).

Chr7: Region 2 (genes 244-268).

*SLG* homolog

*Dw3*

*PLP/OspPLAIIzeta* homolog

K+ transporter*/OsHAK4* homolog

*OsUMAMIT12*/*WAT1* homolog

*BHLH* protein

*ZFHD2*

*MATE* efflux family protein

*CHS*

*SAUR36*

**Chromosome 8:** 3 QTL, 4 genes (269-272).

**Chromosome 9:** 5 QTL, 7 genes (273-279).

**Chromosome 10:** 3 QTL, 5 genes (280-284).
