# Supplementary material for: Dissection of canopy layer-specific genetic control of leaf angle in Sorghum bicolor by RNA sequencing
Source: BMC Genomics. 2022 Feb 3;23:95. doi: 10.1186/s12864-021-08251-4 (PMC8812014; doi:10.1186/s12864-021-08251-4)
Supplement: Supplementary file 2 — Additional file 2: Supplementary Figure S2. A-F. Expression patterns for DEGs co-localizing with leaf angle QTL (grouped by functional category). [file 12864_2021_8251_MOESM2_ESM.docx]

**Supplementary Fig.(s) S2.** A-F. Expression patterns for DE genes (FDR<0.05) co-localizing with leaf angle QTL (grouped by functional category). L5-Leaf 5, L8-Leaf 8, PFL-Pre-flag Leaf.

**A**


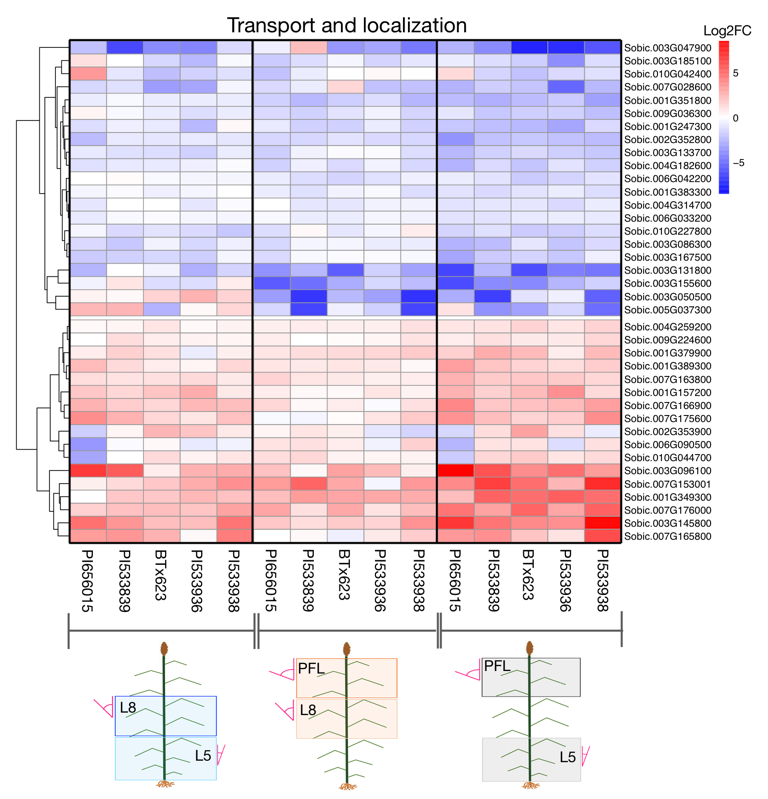


| Gene list: Transport and localization | | |
| --- | --- | --- |
| Sobic.001G157200 | Sobic.003G131800 | Sobic.007G028600 |
| Sobic.001G247300 | Sobic.003G133700 | Sobic.007G153001 |
| Sobic.001G349300 | Sobic.003G145800 | Sobic.007G163800 |
| Sobic.001G351800 | Sobic.003G155600 | Sobic.007G165800 |
| Sobic.001G379900 | Sobic.003G167500 | Sobic.007G166900 |
| Sobic.001G383300 | Sobic.003G185100 | Sobic.007G175600 |
| Sobic.001G389300 | Sobic.004G182600 | Sobic.007G176000 |
| Sobic.002G352800 | Sobic.004G259200 | Sobic.009G036300 |
| Sobic.002G353900 | Sobic.004G314700 | Sobic.009G224600 |
| Sobic.003G047900 | Sobic.005G037300 | Sobic.010G042400 |
| Sobic.003G050500 | Sobic.006G033200 | Sobic.010G044700 |
| Sobic.003G086300 | Sobic.006G042200 | Sobic.010G227800 |
| Sobic.003G096100 | Sobic.006G090500 |  |

Supplementary Fig. S2A

**B**

**
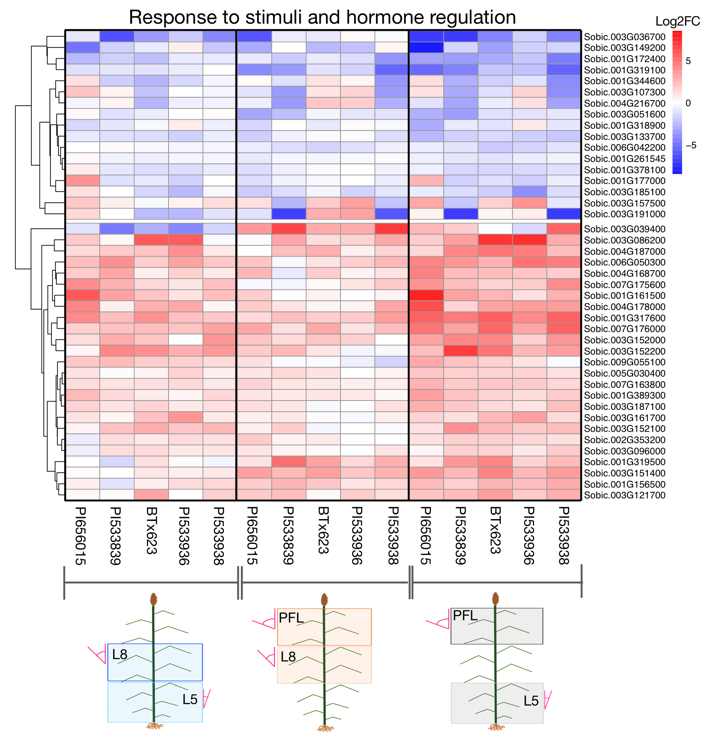
**

| Gene list: Response to stimuli and hormone regulation | | |
| --- | --- | --- |
| Sobic.001G156500 | Sobic.003G039400 | Sobic.003G185100 |
| Sobic.001G161500 | Sobic.003G051600 | Sobic.003G187100 |
| Sobic.001G172400 | Sobic.003G086200 | Sobic.003G191000 |
| Sobic.001G177000 | Sobic.003G096000 | Sobic.004G168700 |
| Sobic.001G261545 | Sobic.003G107300 | Sobic.004G178000 |
| Sobic.001G317600 | Sobic.003G121700 | Sobic.004G187000 |
| Sobic.001G318900 | Sobic.003G133700 | Sobic.004G216700 |
| Sobic.001G319100 | Sobic.003G149200 | Sobic.005G030400 |
| Sobic.001G319500 | Sobic.003G151400 | Sobic.006G042200 |
| Sobic.001G344600 | Sobic.003G152000 | Sobic.006G050300 |
| Sobic.001G378100 | Sobic.003G152100 | Sobic.007G163800 |
| Sobic.001G389300 | Sobic.003G152200 | Sobic.007G175600 |
| Sobic.002G353200 | Sobic.003G157500 | Sobic.007G176000 |
| Sobic.003G036700 | Sobic.003G161700 | Sobic.009G055100 |

Supplementary Fig. S2B

**C**


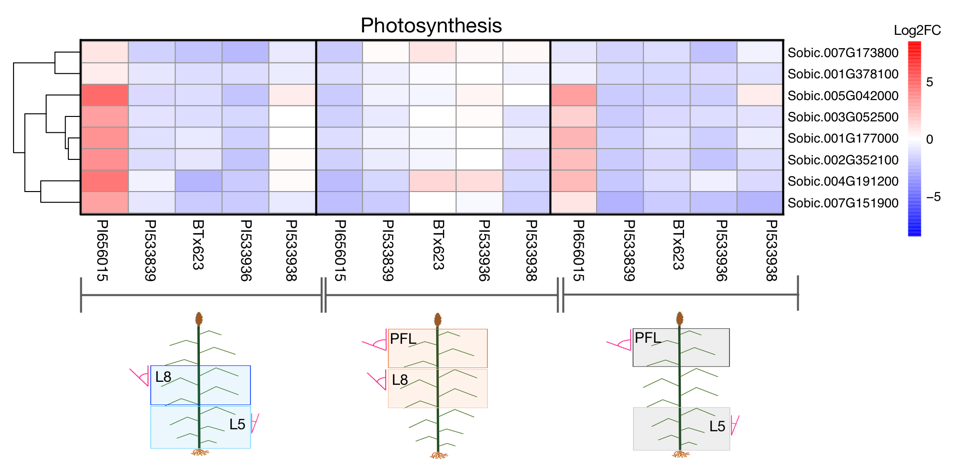


| Gene list: Photosynthesis |
| --- |
| Sobic.001G177000 |
| Sobic.002G352100 |
| Sobic.004G191200 |
| Sobic.007G151900 |
| Sobic.007G173800 |
| Sobic.001G378100 |
| Sobic.003G052500 |
| Sobic.005G042000 |

Supplementary Fig. S2C

**D**


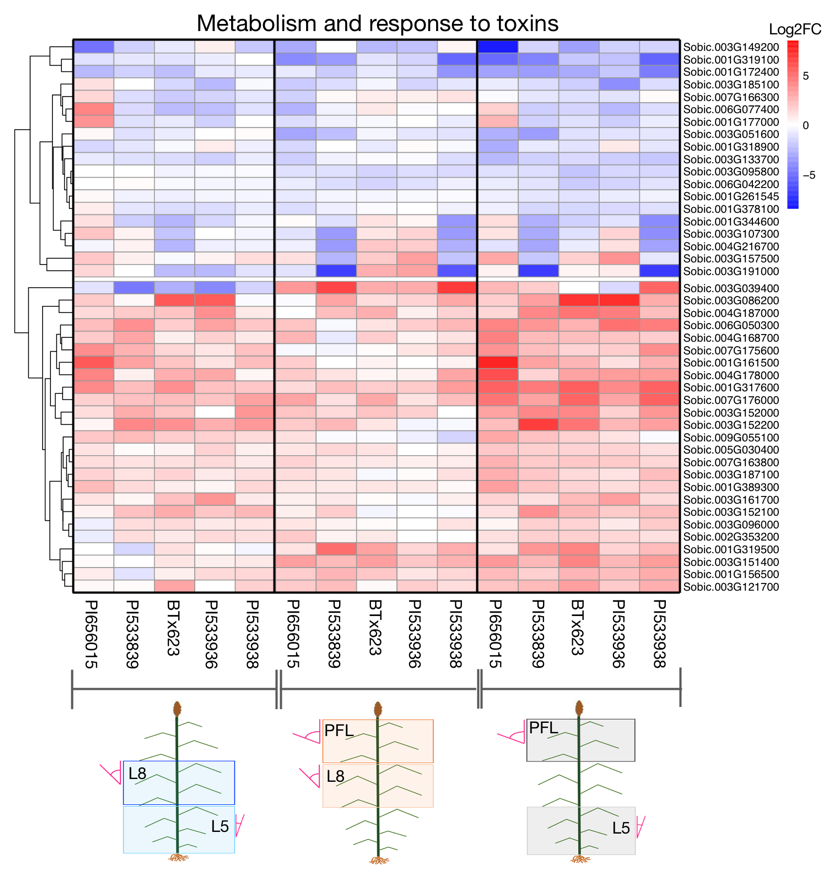


| Gene list: Metabolism and response to toxins | | |
| --- | --- | --- |
| Sobic.001G317600 | Sobic.001G389300 | Sobic.003G107300 |
| Sobic.001G318900 | Sobic.003G151400 | Sobic.003G121700 |
| Sobic.001G319100 | Sobic.003G157500 | Sobic.003G133700 |
| Sobic.001G319500 | Sobic.003G161700 | Sobic.003G149200 |
| Sobic.003G187100 | Sobic.004G168700 | Sobic.003G185100 |
| Sobic.003G152000 | Sobic.007G176000 | Sobic.003G191000 |
| Sobic.003G152100 | Sobic.001G161500 | Sobic.004G178000 |
| Sobic.003G152200 | Sobic.001G172400 | Sobic.004G187000 |
| Sobic.009G055100 | Sobic.001G261545 | Sobic.004G216700 |
| Sobic.003G095800 | Sobic.001G344600 | Sobic.005G030400 |
| Sobic.007G166300 | Sobic.001G378100 | Sobic.006G042200 |
| Sobic.003G096000 | Sobic.002G353200 | Sobic.006G050300 |
| Sobic.006G077400 | Sobic.003G039400 | Sobic.007G163800 |
| Sobic.001G156500 | Sobic.003G051600 | Sobic.007G175600 |
| Sobic.001G177000 | Sobic.003G086200 |  |

Supplementary Fig. S2D

**E**


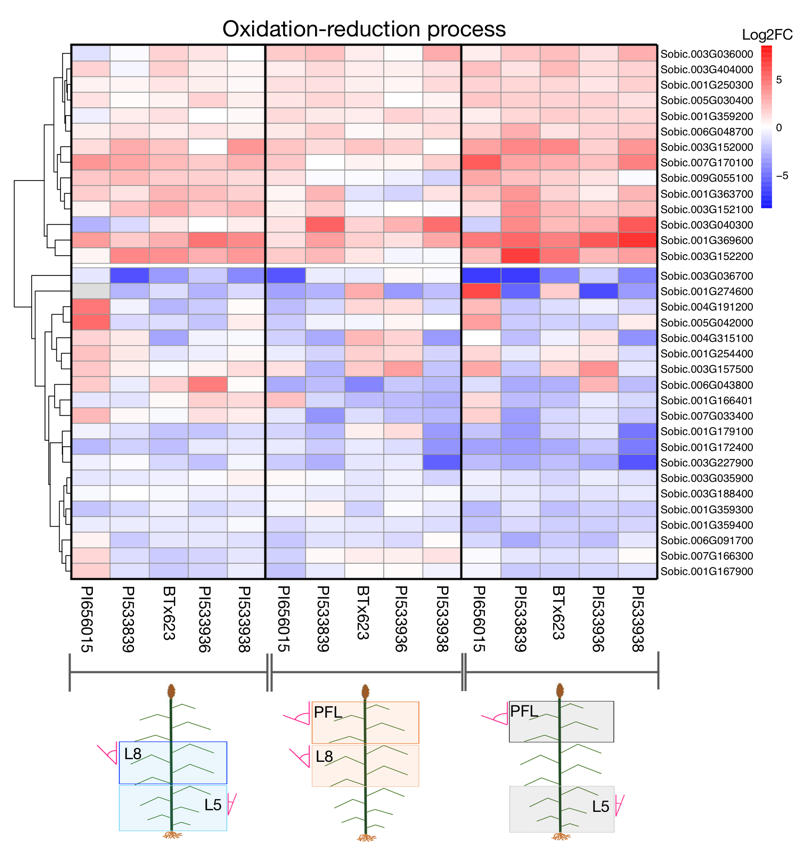


| Gene list: Oxidation-reduction process | | |
| --- | --- | --- |
| Sobic.001G179100 | Sobic.001G369600 | Sobic.004G191200 |
| Sobic.007G166300 | Sobic.003G035900 | Sobic.004G315100 |
| Sobic.001G166401 | Sobic.003G036000 | Sobic.005G030400 |
| Sobic.001G167900 | Sobic.003G036700 | Sobic.005G042000 |
| Sobic.001G172400 | Sobic.003G040300 | Sobic.006G043800 |
| Sobic.001G250300 | Sobic.003G152000 | Sobic.006G048700 |
| Sobic.001G254400 | Sobic.003G152100 | Sobic.006G091700 |
| Sobic.001G274600 | Sobic.003G152200 | Sobic.007G033400 |
| Sobic.001G359200 | Sobic.003G157500 | Sobic.007G170100 |
| Sobic.001G359300 | Sobic.003G188400 | Sobic.009G055100 |
| Sobic.001G359400 | Sobic.003G227900 |  |
| Sobic.001G363700 | Sobic.003G404000 |  |

Supplementary Fig. S2E

**F**


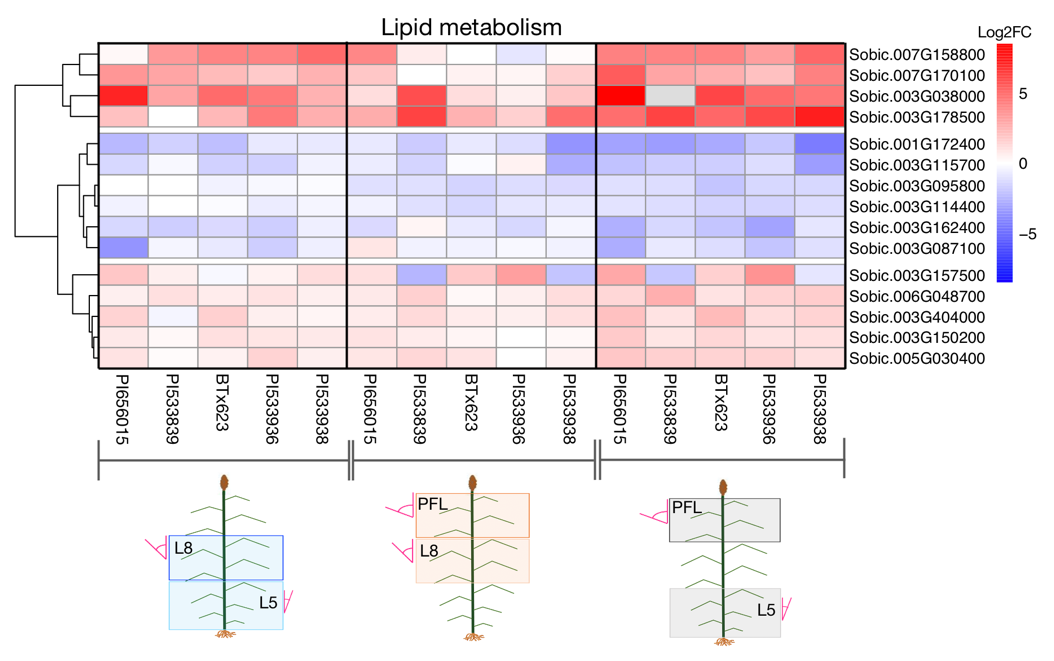


| Gene list: Lipid metabolism | |
| --- | --- |
| Sobic.001G172400 | Sobic.003G115700 |
| Sobic.003G150200 | Sobic.003G178500 |
| Sobic.003G157500 | Sobic.003G404000 |
| Sobic.003G162400 | Sobic.005G030400 |
| Sobic.003G038000 | Sobic.006G048700 |
| Sobic.003G087100 | Sobic.007G158800 |
| Sobic.003G095800 | Sobic.007G170100 |
| Sobic.003G114400 |  |

Supplementary Fig. S2F
