# Supplementary material for: Dissection of canopy layer-specific genetic control of leaf angle in Sorghum bicolor by RNA sequencing
Source: BMC Genomics. 2022 Feb 3;23:95. doi: 10.1186/s12864-021-08251-4 (PMC8812014; doi:10.1186/s12864-021-08251-4)

**Supplementary Fig. S3.** Heatmap summarizing expression patterns for genes of the liguleless family *Sobic.006G247700* (*LG1*), *Sobic.003G363600* (*LG2*), *Sobic.003G144200* (*LG3*), *Sobic.009G030200* (*LG4*) and *Sobic.010G137400* (*LGN-R*). n.s.: non-significant. * FDR<0.05; ** FDR<0.01; *** FDR<0.001.


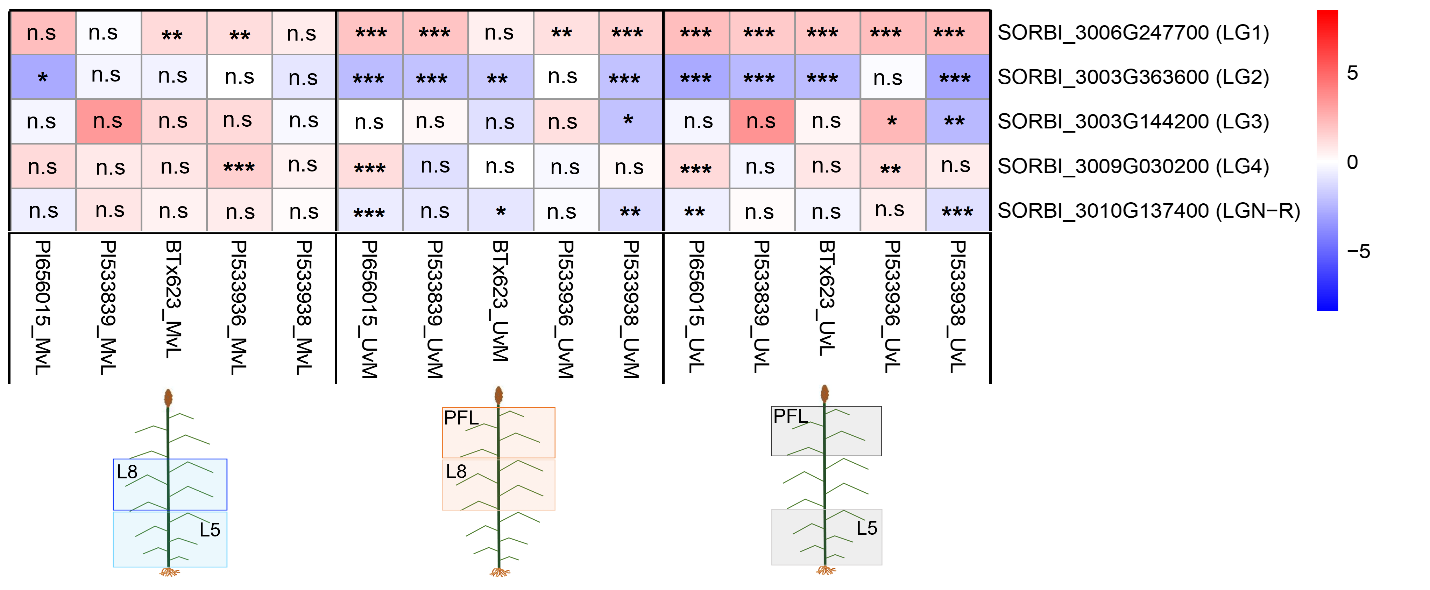

Supplement: Supplementary file 3 — Additional file 3: Supplementary Figure S3. Heatmap summarizing expression patterns of genes of the liguleless family Sobic.006G247700 (LG1), Sobic.003G363600 (LG2), Sobic.003G144200 (LG3), Sobic.009G030200 (LG4) and Sobic.010G137400 (LGN-R). [file 12864_2021_8251_MOESM3_ESM.docx]
