# Supplementary material for: Dissection of canopy layer-specific genetic control of leaf angle in Sorghum bicolor by RNA sequencing
Source: BMC Genomics. 2022 Feb 3;23:95. doi: 10.1186/s12864-021-08251-4 (PMC8812014; doi:10.1186/s12864-021-08251-4)

**Supplementary Fig. S4.** *Sobic.007G175600* (*SAUR36*) and interacting genes. Source: Sorghum FDB (Tian *et al*., 2016). *SAUR36* interacts with *Sobic.009G040700*-BRI1 suppressor 1 (*BSU1*)-like 1 marked with an asterisk.

**
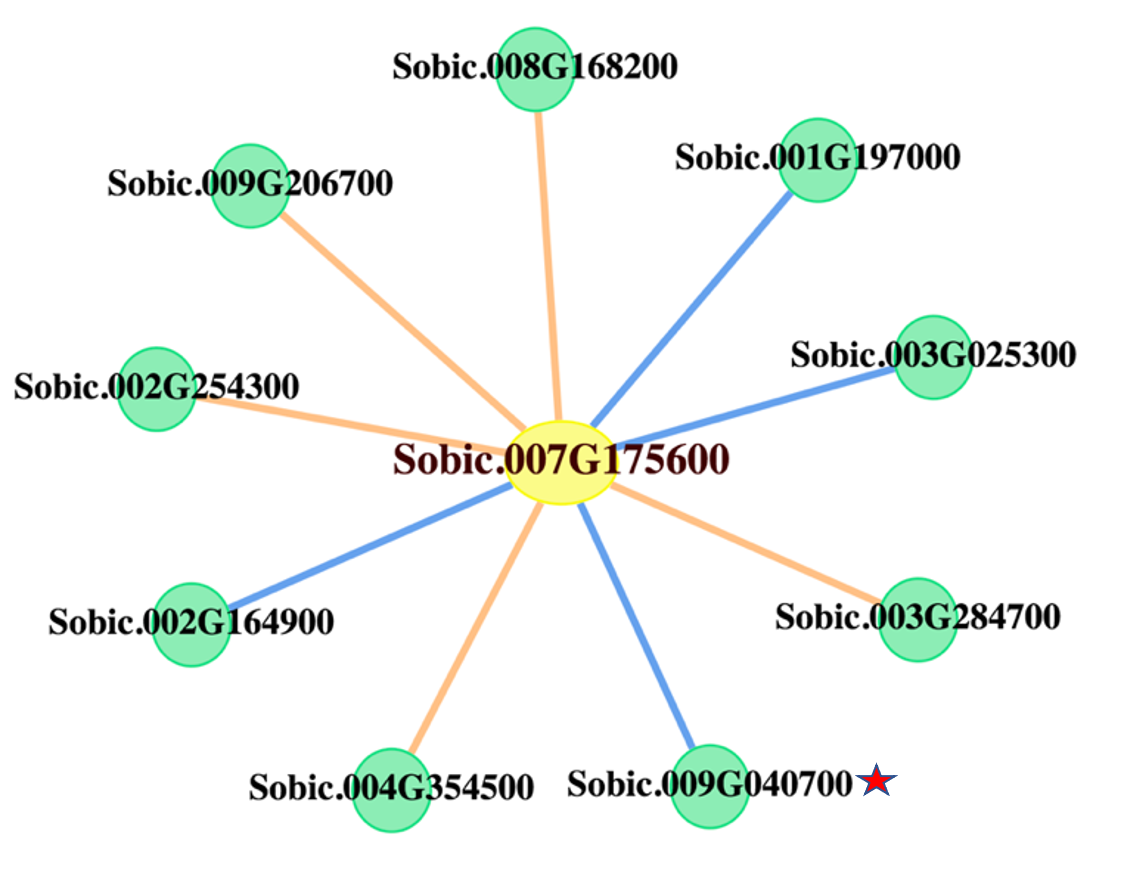
**


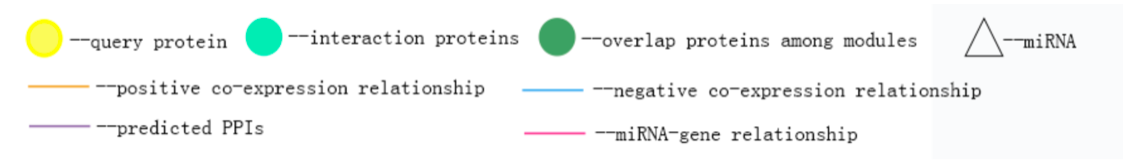

Supplement: Supplementary file 4 — Additional file 4: Supplementary Figure S4. Sobic.007G175600 (SAUR36) and interacting genes. Source: Sorghum FDB (Tian et al., 2016). SAUR36 interacts with Sobic.009G040700-BRI1 suppressor 1 (BSU1)-like 1 marked with an asterisk. [file 12864_2021_8251_MOESM4_ESM.docx]
