# Supplementary material for: Dissection of canopy layer-specific genetic control of leaf angle in Sorghum bicolor by RNA sequencing
Source: BMC Genomics. 2022 Feb 3;23:95. doi: 10.1186/s12864-021-08251-4 (PMC8812014; doi:10.1186/s12864-021-08251-4)

**Supplementary Figure S5.** Collar tissue from sorghum plant as an illustration of the tissue used for RNA extraction.


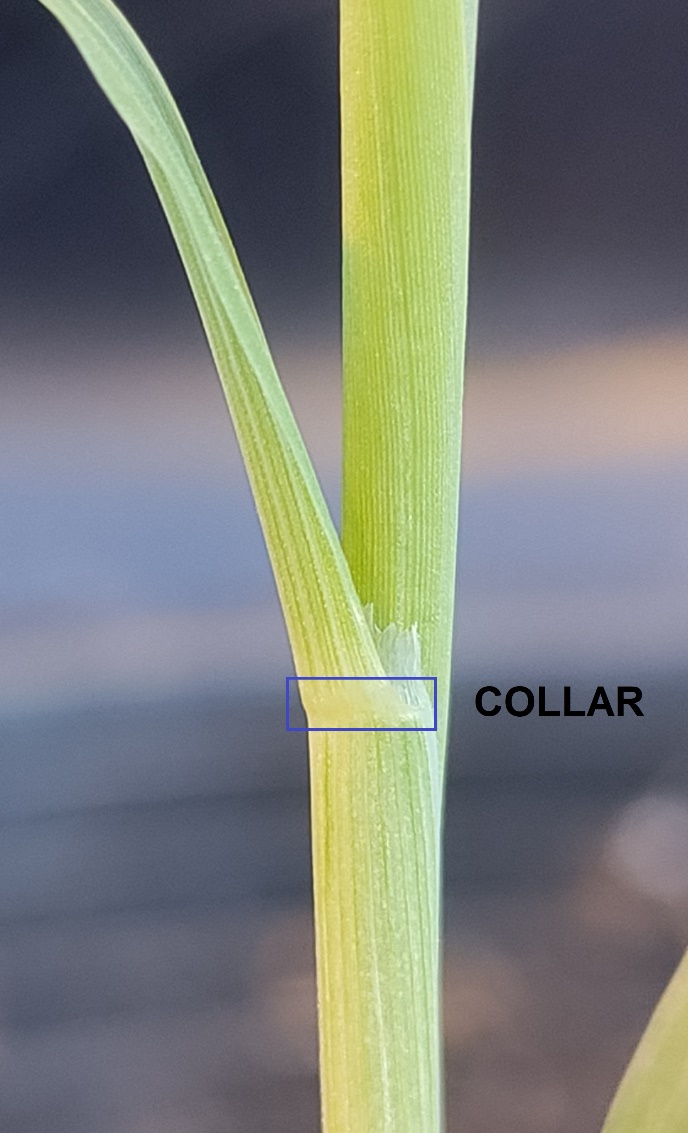

Supplement: Supplementary file 5 — Additional file 5: Supplementary Figure S5. Collar tissue from sorghum plant as an illustration of the tissue used for RNA extraction. [file 12864_2021_8251_MOESM5_ESM.docx]
