# Supplementary figures and images for: Dissection of canopy layer-specific genetic control of leaf angle in Sorghum bicolor by RNA sequencing
Source: BMC Genomics. 2022 Feb 3;23:95. doi: 10.1186/s12864-021-08251-4 (PMC8812014; doi:10.1186/s12864-021-08251-4)

**Supplementary Figure S6.** RNA-seq pipeline used for the study.


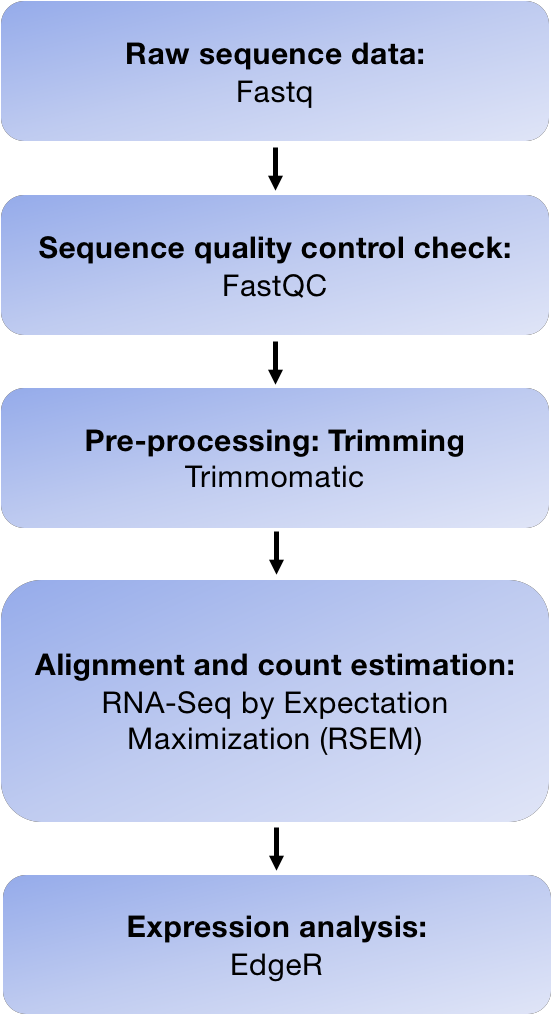

Supplement: Supplementary file 6 — Additional file 6: Supplementary Figure S6. RNA-seq pipeline used for the study. [file 12864_2021_8251_MOESM6_ESM.docx]
