# Supplementary material for: Dissection of canopy layer-specific genetic control of leaf angle in Sorghum bicolor by RNA sequencing
Source: BMC Genomics. 2022 Feb 3;23:95. doi: 10.1186/s12864-021-08251-4 (PMC8812014; doi:10.1186/s12864-021-08251-4)
Supplement: Supplementary file 7 — Additional file 7: Supplementary Table S1. RNA-seq data summary. [file 12864_2021_8251_MOESM7_ESM.docx]

**Supplementary Table S1.** RNA-seq data summary.

| **Sorghum genotype** | **Sample** | **Raw reads** | **Clean reads (trimmed)** | **Overall alignment rate (%)** |
| --- | --- | --- | --- | --- |
| PI656015 | 15C5R2 | 19476571 | 19312476 | 86.2 |
|  | 15C5R3 | 28870602 | 28473917 | 86.3 |
|  | 15C8R1 | 20906329 | 20579722 | 85.9 |
|  | 15C8R2 | 28507904 | 27503199 | 87.0 |
|  | 15C8R3 | 22290294 | 21981481 | 86.6 |
|  | 15CPFLR1 | 16090515 | 15736695 | 87.0 |
|  | 15CPFLR2 | 26690530 | 26536372 | 86.8 |
|  | 15CPFLR3 | 12525455 | 11927690 | 86.8 |
| PI533839 | 39C5R1 | 23730899 | 23582115 | 81.5 |
|  | 39C5R3 | 35536341 | 34810387 | 84.4 |
|  | 39C8R2 | 27416376 | 27098091 | 84.2 |
|  | 39C8R3 | 15511515 | 15099098 | 82.9 |
|  | 39CPFLR1 | 30194002 | 29544312 | 84.0 |
|  | 39CPFLR2 | 26092351 | 25859214 | 84.8 |
|  | 39CPFLR3 | 24147738 | 23186956 | 85.0 |
| BTx623 | TxC5R1 | 21137158 | 21003098 | 88.9 |
|  | TxC5R2 | 22268292 | 21969473 | 88.0 |
|  | TxC8R2 | 24526382 | 24374160 | 88.9 |
|  | TxC8R3 | 25769542 | 25002243 | 90.0 |
|  | TxCPFLR1 | 31837040 | 31555084 | 89.4 |
|  | TxCPFLR2 | 24732412 | 24592824 | 88.5 |
|  | TxCPFLR3 | 24696238 | 24391091 | 90.0 |
| PI533936 | 36C5R2 | 24547618 | 24467297 | 86.3 |
|  | 36C5R3 | 22080730 | 21807124 | 86.8 |
|  | 36C8R1 | 16447599 | 16286523 | 86.7 |
|  | 36C8R2 | 28373732 | 28037509 | 87.1 |
|  | 36C8R3 | 17572117 | 17396293 | 86.5 |
|  | 36CPFLR1 | 16665727 | 15539442 | 80.7 |
|  | 36CPFLR2 | 19230329 | 19063913 | 85.7 |
|  | 36CPFLR3 | 28628711 | 28143655 | 85.4 |
| PI533938 | 38C5R2 | 22689855 | 22568072 | 85.2 |
|  | 38C5R3 | 24555024 | 23347250 | 86.2 |
|  | 38C8R1 | 20165238 | 20009024 | 86.0 |
|  | 38C8R2 | 24042599 | 23754540 | 86.1 |
|  | 38C8R3 | 25534506 | 25281235 | 86.0 |
|  | 38CPFLR1 | 28735305 | 27799663 | 77.3 |
|  | 38CPFLR2 | 28123990 | 27290307 | 78.2 |
|  | 38CPFLR3 | 16528003 | 15298396 | 79.3 |
|  | **Sum** | **896875569** | **880209941** |  |
