# Supplementary material for: Dissection of canopy layer-specific genetic control of leaf angle in Sorghum bicolor by RNA sequencing
Source: BMC Genomics. 2022 Feb 3;23:95. doi: 10.1186/s12864-021-08251-4 (PMC8812014; doi:10.1186/s12864-021-08251-4)
Supplement: Supplementary file 8 — Additional file 8: Supplementary Table S2. Sorghum leaf angle QTL (69), number of DEGs co-localizing with QTL on each chromosome, and references. [file 12864_2021_8251_MOESM8_ESM.docx]

**Supplementary Table S2.** Sorghum leaf angle QTL (69), number of DEGs co-localizing with QTL on each chromosome, and references.

| **QTL** | **LG:Start-End (v3.0)** | **Genes Under QTL (v3.0)** | **Number of DEGs co-localizing with QTL** | **Reference** |
| --- | --- | --- | --- | --- |
| QLANG1.8 | 1:12498949-14699779 | 201 | 68 | Hart *et al*., 2001 |
| QLANG1.6 | 1:12504838-15806148 | 305 |  | Truong *et al*., 2015 |
| QLANG1.5 | 1:24054104-53206564 | 438 |  | Truong *et al*., 2015 |
| QLANG1.4 | 1:24111042-53154261 | 434 |  | Truong *et al*., 2015 |
| QLANG1.1 | 1:54369359-54929968 | 16 |  | Zhao *et al*., 2016 |
| QLANG1.9 | 1:59056306-59176541 | 7 |  | Mantilla Perez *et al*., 2014 |
| QLANG1.7 | 1:60544139-61389455 | 103 |  | Truong *et al*., 2015 |
| QLANG1.3 | 1:60950563-61601617 | 61 |  | Truong *et al*., 2015 |
| QLANG1.2 | 1:64605704-67693590 | 346 |  | Truong *et al*., 2015 |
| qP3-L4/L5-1.1 | 1:63335734-64409573 | 107 |  | Mantilla-Perez *et al*., 2020 |
| qP2-L5-1.1 | 1:76023167-76291376 | 38 |  | Mantilla-Perez *et al*., 2020 |
| QLANG2.1 | 2:61967677-62028496 | 11 | 7 | Mantilla Perez *et al*., 2014 |
| QLANG2.2 | 2:71448036-71900721 | 60 |  | Mantilla Perez *et al*., 2014 |
| QLANG2.3 | 2:71448048-71903345 | 60 |  | Mantilla Perez *et al*., 2014 |
| QLANG3.1 | 3:3134407-4422207 | 145 | 99 | Zhao *et al*., 2016 |
| QLANG3.5 | 3:4560802-4884543 | 44 |  | Mantilla Perez *et al*., 2014 |
| QLANG3.6 | 3:7002635-7312470 | 34 |  | Mantilla Perez *et al*., 2014 |
| QLANG3.7 | 3:7110000-11460000 | 443 |  | McCormick *et al*., 2017 |
| QLANG3.2 | 3:14889177-51648272 | 518 |  | Truong *et al*., 2015 |
| QLANG3.3 | 3:55331681-56685744 | 112 |  | Truong *et al*., 2015 |
| QLANG3.4 | 3:57033712-57739838 | 76 |  | Truong *et al*., 2015 |
| qP2-All-3.1 | 3:70281299-71232951 | 143 |  | Mantilla-Perez *et al*., 2020 |
| qP1-PFL-3.1 | 3:8255096-8666433 | 34 |  | Mantilla-Perez *et al*., 2020 |
| qP1-PFL-3.2 | 3:73382999-73445162 | 7 |  | Mantilla-Perez *et al*., 2020 |
| qP1-L4-3.1 | 3:6781489-7045880 | 35 |  | Mantilla-Perez *et al*., 2020 |
| qP1-L4-3.2 | 3:73010518-73027051 | 6 |  | Mantilla-Perez *et al*., 2020 |
| qP1-L5-3.1 | 3:12467433-12784618 | 25 |  | Mantilla-Perez *et al*., 2020 |
| qP3-L5-3.1 | 3:11966122-12932189 | 75 |  | Mantilla-Perez *et al*., 2020 |
| QLANG4.1 | 4:51930000-56730000 | 513 | 28 | McCormick *et al*., 2017 |
| qP2-L4/L5-4.1 | 4:65072994-65249841 | 29 |  | Mantilla-Perez *et al*., 2020 |
| qP1-L4-4.1 | 4:59603083-59721191 | 20 |  | Mantilla-Perez *et al*., 2020 |
| qP1-L5-4.1 | 4:60400711-60538317 | 22 |  | Mantilla-Perez *et al*., 2020 |
| QLANG5.1 | 5:2655041-4865134 | 221 | 14 | Truong *et al*., 2015 |
| qP2-PFL/L4-5.1 | 5:60597128-61546233 | 44 |  | Mantilla-Perez *et al*., 2020 |
| qP3-L4/L5-5.1 | 5:54754816-56100971 | 39 |  | Mantilla-Perez *et al*., 2020 |
| QLANG6.1 | 6:10011933-14194526 | 30 | 22 | Zhao *et al*., 2016 |
| QLANG6.2 | 6:27829682-38481880 | 111 |  | Zhao *et al*., 2016 |
| QLANG6.4 | 6:44002171-46287447 | 179 |  | Hart *et al*., 2001 |
| QLANG6.3 | 6:53742729-53845255 | 8 |  | Zhao *et al.,* 2016 |
| qP2-PFL-6.1 | 6:6051417-38428465 | 263 |  | Mantilla-Perez *et al*., 2020 |
| qP3-PFL-6.1 | 6:44971241-45648729 | 93 |  | Mantilla-Perez *et al*., 2020 |
| QLANG7.8 | 7:57706101-58490454 | 56 | 30 | Truong *et al*., 2015 |
| QLANG7.10 | 7:58480000-61098149 | 254 |  | McCormick *et al*., 2017 |
| QLANG7.4 | 7:59165496-59536406 | 34 |  | Truong *et al*., 2015 |
| QLANG7.13 | 7:59200000-61098149 | 196 |  | McCormick *et al*., 2017 |
| QLANG7.5 | 7:59233126-59537387 | 26 |  | Truong *et al*., 2015 |
| 1.67Mb_GR | 7:59397316-61070995 | 181 |  | Zhao *et al*., 2016 |
| QLANG7.12 | 7:59480000-61098149 | 172 |  | McCormick *et al*., 2017 |
| QLANG7.11 | 7:59510000-61098149 | 171 |  | McCormick *et al*., 2017 |
| QLANG7.7 | 7:59529989-59535099 | 1 |  | Truong *et al*., 2015 |
| QLANG7.6 | 7:59530265-59534822 | 1 |  | Truong *et al*., 2015 |
| QLANG7.9 | 7:59540892-59550021 | 0 |  | Hart *et al*., 2001 |
| qP3-All-7.1 | 7:59785398-59991087 | 20 |  | Mantilla-Perez *et al*., 2020 |
| qP2-PFL/L4-7.1 | 7:59458622-59821657 | 38 |  | Mantilla-Perez *et al*., 2020 |
| qP3-L4/L5-7.1 | 7:60264032-61110866 | 104 |  | Mantilla-Perez *et al*., 2020 |
| qP3-L4/L5-7.2 | 7:59654592-60193797 | 49 |  | Mantilla-Perez *et al*., 2020 |
| qP1-L5-7.1 | 7:2528634-2953302 | 60 |  | Mantilla-Perez *et al*., 2020 |
| qP2-L5-7.2 | 7:60308754-60676001 | 35 |  | Mantilla-Perez *et al*., 2020 |
| qP1-L4/L5-8.1 | 8:3037472-3103413 | 6 | 4 | Mantilla-Perez *et al*., 2020 |
| qP2-L4/L5-8.1 | 8:59212870-59429224 | 21 |  | Mantilla-Perez *et al*., 2020 |
| qP1-PFL-8.1 | 8:3231971-3664861 | 39 |  | Mantilla-Perez *et al*., 2020 |
| QLANG9.1 | 9:1000006-1049867 | 11 | 7 | Zhao *et al*., 2016 |
| qP1-PFL/L4-9.1 | 9:3073655-3346678 | 26 |  | Mantilla-Perez *et al*., 2020 |
| qP2-PFL/L4-9.1 | 9:56458867-56715113 | 38 |  | Mantilla-Perez *et al*., 2020 |
| qP2-PFL-9.1 | 9:5496111-6366929 | 74 |  | Mantilla-Perez *et al*., 2020 |
| qP1-L5-9.1 | 9:2711949-2736192 | 4 |  | Mantilla-Perez *et al*., 2020 |
| qP3-PFL/L4-10.1 | 10:56937398-57191924 | 25 | 5 | Mantilla-Perez *et al*., 2020 |
| qP3-PFL-10.3 | 10:3055149-3626545 | 91 |  | Mantilla-Perez *et al*., 2020 |
| qP3-L4-10.1 | 10:56937398-57191924 | 25 |  | Mantilla-Perez *et al*., 2020 |

Source: Sorghum QTL Atlas (Mace *et al*., 2019) and Ensembl Plants.
