# Supplementary material for: Dissection of canopy layer-specific genetic control of leaf angle in Sorghum bicolor by RNA sequencing
Source: BMC Genomics. 2022 Feb 3;23:95. doi: 10.1186/s12864-021-08251-4 (PMC8812014; doi:10.1186/s12864-021-08251-4)
Supplement: Supplementary file 9 — Additional file 9: Supplementary Table S3. Differentially expressed genes co-localizing with leaf angle QTL (shown in Supplementary Fig. S2 graphs). [file 12864_2021_8251_MOESM9_ESM.docx]

**Supplementary Table S3.** Differentially expressed genes co-localizing with leaf angle QTL (shown in Supplementary Fig. S2 graphs).

| **Chromosome** | **Gene_id** | **Bp_start** | **Bp_end** | **Number in graph** |
| --- | --- | --- | --- | --- |
| Chromosome 1 | Sobic.001G156500 | 12656287 | 12658943 | 1 |
|  | Sobic.001G157200 | 12708627 | 12713276 | 2 |
|  | Sobic.001G158000 | 12813999 | 12819113 | 3 |
|  | Sobic.001G158900 | 12928101 | 12936071 | 4 |
|  | Sobic.001G160200 | 13149779 | 13157433 | 5 |
|  | Sobic.001G161500 | 13278084 | 13279390 | 6 |
|  | Sobic.001G166401 | 13838450 | 13840200 | 7 |
|  | Sobic.001G167900 | 13971059 | 13986280 | 8 |
|  | Sobic.001G170301 | 14206452 | 14208245 | 9 |
|  | Sobic.001G172400 | 14434718 | 14438560 | 10 |
|  | Sobic.001G177000 | 14892135 | 14893503 | 11 |
|  | Sobic.001G178250 | 15011120 | 15013043 | 12 |
|  | Sobic.001G179100 | 15159551 | 15161682 | 13 |
|  | Sobic.001G180200 | 15278638 | 15284275 | 14 |
|  | Sobic.001G237700 | 24337515 | 24344462 | 15 |
|  | Sobic.001G241200 | 24822042 | 24830476 | 16 |
|  | Sobic.001G247300 | 26238940 | 26241978 | 17 |
|  | Sobic.001G250300 | 27003145 | 27011465 | 18 |
|  | Sobic.001G251400 | 27152151 | 27157196 | 19 |
|  | Sobic.001G254400 | 28476069 | 28479416 | 20 |
|  | Sobic.001G258200 | 30649723 | 30655105 | 21 |
|  | Sobic.001G258300 | 30661673 | 30663370 | 22 |
|  | Sobic.001G261545 | 38159812 | 38181673 | 23 |
|  | Sobic.001G261549 | 38192629 | 38196798 | 24 |
|  | Sobic.001G263100 | 47286071 | 47294460 | 25 |
|  | Sobic.001G267800 | 51092090 | 51094503 | 26 |
|  | Sobic.001G274600 | 53159900 | 53161867 | 27 |
|  | Sobic.001G274700 | 53165615 | 53167762 | 28 |
|  | Sobic.001G317600 | 60581424 | 60582916 | 29 |
|  | Sobic.001G318900 | 60702173 | 60703732 | 30 |
|  | Sobic.001G319100 | 60717647 | 60718433 | 31 |
|  | Sobic.001G319500 | 60742198 | 60743267 | 32 |
|  | Sobic.001G320000 | 60784896 | 60788922 | 33 |
|  | Sobic.001G323701 | 61111829 | 61116329 | 34 |
|  | Sobic.001G324700 | 61198278 | 61199489 | 35 |
|  | Sobic.001G324800 | 61201114 | 61201921 | 36 |
|  | Sobic.001G326200 | 61335681 | 61336584 | 37 |
|  | Sobic.001G344600 | 63335368 | 63336120 | 38 |
|  | Sobic.001G349300 | 63890519 | 63892946 | 39 |
|  | Sobic.001G351100 | 64072396 | 64074291 | 40 |
|  | Sobic.001G351200 | 64076006 | 64078731 | 41 |
|  | Sobic.001G351800 | 64121222 | 64123966 | 42 |
|  | Sobic.001G353300 | 64288863 | 64291248 | 43 |
|  | Sobic.001G353400 | 64352146 | 64357974 | 44 |
|  | Sobic.001G359200 | 64901749 | 64904745 | 45 |
|  | Sobic.001G359300 | 64908610 | 64911800 | 46 |
|  | Sobic.001G359400 | 64930433 | 64933173 | 47 |
|  | Sobic.001G359700 | 64944686 | 64945267 | 48 |
|  | Sobic.001G363700 | 65296362 | 65298761 | 49 |
|  | Sobic.001G368600 | 65730425 | 65734584 | 50 |
|  | Sobic.001G368900 | 65739329 | 65742422 | 51 |
|  | Sobic.001G369401 | 65800098 | 65803214 | 52 |
|  | Sobic.001G369600 | 65822985 | 65828764 | 53 |
|  | Sobic.001G369701 | 65829517 | 65830411 | 54 |
|  | Sobic.001G375800 | 66414676 | 66415195 | 55 |
|  | Sobic.001G378100 | 66608981 | 66621756 | 56 |
|  | Sobic.001G378300 | 66642503 | 66650446 | 57 |
|  | Sobic.001G379000 | 66727103 | 66730129 | 58 |
|  | Sobic.001G379900 | 66813262 | 66819613 | 59 |
|  | Sobic.001G381500 | 66951662 | 66956362 | 60 |
|  | Sobic.001G382400 | 67013756 | 67018873 | 61 |
|  | Sobic.001G383300 | 67079510 | 67094300 | 62 |
|  | Sobic.001G385900 | 67327321 | 67329183 | 63 |
|  | Sobic.001G388800 | 67551766 | 67554569 | 64 |
|  | Sobic.001G389300 | 67599333 | 67603108 | 65 |
|  | Sobic.001G389400 | 67607317 | 67610403 | 66 |
|  | Sobic.001G491900 | 76156637 | 76159527 | 67 |
|  | Sobic.001G492000 | 76161220 | 76162658 | 68 |
| Chromosome 2 | Sobic.002G228000 | 61968598 | 61970330 | 69 |
|  | Sobic.002G228400 | 61981349 | 61984595 | 70 |
|  | Sobic.002G351200 | 71510043 | 71514525 | 71 |
|  | Sobic.002G352100 | 71556302 | 71558107 | 72 |
|  | Sobic.002G353200 | 71635214 | 71637226 | 73 |
|  | Sobic.002G353900 | 71733857 | 71736165 | 74 |
|  | Sobic.002G352800 | 71596493 | 71603554 | 75 |
| Chromosome 3 | Sobic.003G035900 | 3287186 | 3289168 | 76 |
|  | Sobic.003G036000 | 3299322 | 3305357 | 77 |
|  | Sobic.003G036700 | 3381538 | 3384118 | 78 |
|  | Sobic.003G037300 | 3504460 | 3508169 | 79 |
|  | Sobic.003G038000 | 3570808 | 3573716 | 80 |
|  | Sobic.003G039400 | 3695196 | 3696666 | 81 |
|  | Sobic.003G040300 | 3745527 | 3750862 | 82 |
|  | Sobic.003G042900 | 3966341 | 3968202 | 83 |
|  | Sobic.003G045100 | 4133481 | 4136435 | 84 |
|  | Sobic.003G045300 | 4142665 | 4151071 | 85 |
|  | Sobic.003G047900 | 4375572 | 4381767 | 86 |
|  | Sobic.003G050000 | 4571153 | 4572861 | 87 |
|  | Sobic.003G050500 | 4593264 | 4594236 | 88 |
|  | Sobic.003G051600 | 4667295 | 4668993 | 89 |
|  | Sobic.003G052000 | 4681151 | 4686500 | 90 |
|  | Sobic.003G052500 | 4731408 | 4732609 | 91 |
|  | Sobic.003G079300 | 6804540 | 6807751 | 92 |
|  | Sobic.003G081100 | 6981610 | 6986283 | 93 |
|  | Sobic.003G082300 | 7065028 | 7066220 | 94 |
|  | Sobic.003G082600 | 7081834 | 7084517 | 95 |
|  | Sobic.003G084200 | 7229152 | 7235363 | 96 |
|  | Sobic.003G085400 | 7355471 | 7356700 | 97 |
|  | Sobic.003G085600 | 7371175 | 7373163 | 98 |
|  | Sobic.003G086200 | 7454836 | 7455699 | 99 |
|  | Sobic.003G086300 | 7462786 | 7470186 | 100 |
|  | Sobic.003G087100 | 7549923 | 7551848 | 101 |
|  | Sobic.003G092500 | 8039860 | 8050143 | 102 |
|  | Sobic.003G095700 | 8404873 | 8408492 | 103 |
|  | Sobic.003G095800 | 8416682 | 8423223 | 104 |
|  | Sobic.003G096000 | 8474356 | 8478421 | 105 |
|  | Sobic.003G096100 | 8499492 | 8501691 | 106 |
|  | Sobic.003G097100 | 8588949 | 8592440 | 107 |
|  | Sobic.003G099400 | 8756213 | 8757624 | 108 |
|  | Sobic.003G102200 | 9069872 | 9073990 | 109 |
|  | Sobic.003G105000 | 9486491 | 9491334 | 110 |
|  | Sobic.003G105500 | 9539637 | 9543843 | 111 |
|  | Sobic.003G107300 | 9713280 | 9715515 | 112 |
|  | Sobic.003G108000 | 9779774 | 9779986 | 113 |
|  | Sobic.003G108200 | 9786854 | 9788272 | 114 |
|  | Sobic.003G108500 | 9796888 | 9798624 | 115 |
|  | Sobic.003G108900 | 9827685 | 9831906 | 116 |
|  | Sobic.003G114100 | 10269403 | 10271290 | 117 |
|  | Sobic.003G114400 | 10289509 | 10296874 | 118 |
|  | Sobic.003G114800 | 10339550 | 10340528 | 119 |
|  | Sobic.003G114900 | 10369907 | 10370413 | 120 |
|  | Sobic.003G115700 | 10427410 | 10431141 | 121 |
|  | Sobic.003G121700 | 11095566 | 11097388 | 122 |
|  | Sobic.003G131300 | 12314277 | 12316714 | 123 |
|  | Sobic.003G131800 | 12374712 | 12381298 | 124 |
|  | Sobic.003G133700 | 12590234 | 12594829 | 125 |
|  | Sobic.003G133800 | 12594355 | 12598756 | 126 |
|  | Sobic.003G135400 | 12853073 | 12857181 | 127 |
|  | Sobic.003G145800 | 14992220 | 14995112 | 128 |
|  | Sobic.003G146300 | 15040130 | 15041878 | 129 |
|  | Sobic.003G149200 | 15747283 | 15748363 | 130 |
|  | Sobic.003G149500 | 15769796 | 15774146 | 131 |
|  | Sobic.003G150200 | 15833855 | 15837585 | 132 |
|  | Sobic.003G150400 | 15849087 | 15850676 | 133 |
|  | Sobic.003G150600 | 15871537 | 15877302 | 134 |
|  | Sobic.003G151100 | 15908238 | 15909569 | 135 |
|  | Sobic.003G151400 | 15952858 | 15956907 | 136 |
|  | Sobic.003G152000 | 16131503 | 16135762 | 137 |
|  | Sobic.003G152100 | 16139803 | 16144509 | 138 |
|  | Sobic.003G152200 | 16165846 | 16168878 | 139 |
|  | Sobic.003G152300 | 16186119 | 16188121 | 140 |
|  | Sobic.003G155100 | 16886062 | 16902570 | 141 |
|  | Sobic.003G155600 | 17099441 | 17102745 | 142 |
|  | Sobic.003G157500 | 17759475 | 17762059 | 143 |
|  | Sobic.003G157700 | 17801832 | 17804049 | 144 |
|  | Sobic.003G160800 | 19148402 | 19153727 | 145 |
|  | Sobic.003G161700 | 19410275 | 19417561 | 146 |
|  | Sobic.003G162400 | 19934543 | 19940549 | 147 |
|  | Sobic.003G164700 | 21131769 | 21132823 | 148 |
|  | Sobic.003G166200 | 24834677 | 24840210 | 149 |
|  | Sobic.003G167500 | 27265060 | 27271626 | 150 |
|  | Sobic.003G173950 | 41122694 | 41125902 | 151 |
|  | Sobic.003G174700 | 42068917 | 42072321 | 152 |
|  | Sobic.003G178500 | 45640683 | 45642479 | 153 |
|  | Sobic.003G185100 | 49714573 | 49717493 | 154 |
|  | Sobic.003G185400 | 49904688 | 49906730 | 155 |
|  | Sobic.003G187100 | 50578796 | 50580673 | 156 |
|  | Sobic.003G188400 | 50795712 | 50801094 | 157 |
|  | Sobic.003G191000 | 51236002 | 51237593 | 158 |
|  | Sobic.003G191600 | 51265638 | 51266746 | 159 |
|  | Sobic.003G191800 | 51321805 | 51324198 | 160 |
|  | Sobic.003G220100 | 55532134 | 55537475 | 161 |
|  | Sobic.003G226200 | 56261930 | 56268099 | 162 |
|  | Sobic.003G226300 | 56272611 | 56276329 | 163 |
|  | Sobic.003G226800 | 56376690 | 56378642 | 164 |
|  | Sobic.003G227900 | 56543055 | 56548425 | 165 |
|  | Sobic.003G232100 | 57142469 | 57145548 | 166 |
|  | Sobic.003G233100 | 57209172 | 57215752 | 167 |
|  | Sobic.003G235300 | 57367678 | 57370325 | 168 |
|  | Sobic.003G235500 | 57383232 | 57383791 | 169 |
|  | Sobic.003G394400 | 70519047 | 70520318 | 170 |
|  | Sobic.003G397300 | 70710569 | 70711410 | 171 |
|  | Sobic.003G397600 | 70721310 | 70722503 | 172 |
|  | Sobic.003G397700 | 70722504 | 70725550 | 173 |
|  | Sobic.003G404000 | 71173869 | 71178692 | 174 |
| Chromosome 4 | Sobic.004G168700 | 51949746 | 51951465 | 175 |
|  | Sobic.004G170600 | 52319194 | 52324272 | 176 |
|  | Sobic.004G176200 | 52851000 | 52859491 | 177 |
|  | Sobic.004G178000 | 53045938 | 53049453 | 178 |
|  | Sobic.004G179200 | 53210626 | 53215123 | 179 |
|  | Sobic.004G180200 | 53279614 | 53282637 | 180 |
|  | Sobic.004G182600 | 53645079 | 53646910 | 181 |
|  | Sobic.004G185200 | 53801359 | 53803654 | 182 |
|  | Sobic.004G186600 | 53889452 | 53891855 | 183 |
|  | Sobic.004G187000 | 53915182 | 53919172 | 184 |
|  | Sobic.004G190700 | 54272692 | 54277680 | 185 |
|  | Sobic.004G191200 | 54308040 | 54309290 | 186 |
|  | Sobic.004G197000 | 54809282 | 54810114 | 187 |
|  | Sobic.004G197100 | 54826406 | 54827277 | 188 |
|  | Sobic.004G201300 | 55299576 | 55302358 | 189 |
|  | Sobic.004G207200 | 55759003 | 55765496 | 190 |
|  | Sobic.004G208900 | 55861658 | 55866882 | 191 |
|  | Sobic.004G211501 | 56120011 | 56123296 | 192 |
|  | Sobic.004G212800 | 56246907 | 56252442 | 193 |
|  | Sobic.004G214000 | 56379572 | 56384591 | 194 |
|  | Sobic.004G215800 | 56565003 | 56569173 | 195 |
|  | Sobic.004G216700 | 56625893 | 56628817 | 196 |
|  | Sobic.004G250200 | 59678091 | 59681244 | 197 |
|  | Sobic.004G258500 | 60408405 | 60410179 | 198 |
|  | Sobic.004G259200 | 60460359 | 60464051 | 199 |
|  | Sobic.004G314700 | 65118864 | 65128829 | 200 |
|  | Sobic.004G314800 | 65130903 | 65131484 | 201 |
|  | Sobic.004G315100 | 65155968 | 65158224 | 202 |
| Chromosome 5 | Sobic.005G030300 | 2693372 | 2697671 | 203 |
|  | Sobic.005G030400 | 2705723 | 2709254 | 204 |
|  | Sobic.005G032400 | 2892613 | 2896122 | 205 |
|  | Sobic.005G034000 | 3065482 | 3070311 | 206 |
|  | Sobic.005G034100 | 3079494 | 3083070 | 207 |
|  | Sobic.005G036600 | 3335433 | 3340814 | 208 |
|  | Sobic.005G037300 | 3408590 | 3413444 | 209 |
|  | Sobic.005G042000 | 3876057 | 3877378 | 210 |
|  | Sobic.005G046500 | 4393842 | 4407287 | 211 |
|  | Sobic.005G047100 | 4469547 | 4472327 | 212 |
|  | Sobic.005G126300 | 55036181 | 55040015 | 213 |
|  | Sobic.005G129400 | 56035760 | 56042261 | 214 |
|  | Sobic.005G142800 | 60630738 | 60651145 | 215 |
|  | Sobic.005G142900 | 60659170 | 60662154 | 216 |
| Chromosome 6 | Sobic.006G028800 | 6055955 | 6064517 | 217 |
|  | Sobic.006G029550 | 6216681 | 6221173 | 218 |
|  | Sobic.006G032700 | 7357701 | 7362253 | 219 |
|  | Sobic.006G033200 | 8035812 | 8049585 | 220 |
|  | Sobic.006G037700 | 14487572 | 14548530 | 221 |
|  | Sobic.006G039100 | 16726714 | 16727398 | 222 |
|  | Sobic.006G042200 | 28152845 | 28164202 | 223 |
|  | Sobic.006G043800 | 30398857 | 30401232 | 224 |
|  | Sobic.006G045000 | 31573923 | 31574312 | 225 |
|  | Sobic.006G046000 | 32166386 | 32167991 | 226 |
|  | Sobic.006G046600 | 32287957 | 32294195 | 227 |
|  | Sobic.006G048700 | 34559165 | 34562646 | 228 |
|  | Sobic.006G050300 | 37701935 | 37704246 | 229 |
|  | Sobic.006G050500 | 37777620 | 37787490 | 230 |
|  | Sobic.006G076000 | 44064996 | 44066356 | 231 |
|  | Sobic.006G077400 | 44359730 | 44363756 | 232 |
|  | Sobic.006G080800 | 44957353 | 44961057 | 233 |
|  | Sobic.006G086000 | 45545065 | 45546863 | 234 |
|  | Sobic.006G090500 | 46063577 | 46072140 | 235 |
|  | Sobic.006G091700 | 46186783 | 46189648 | 236 |
|  | Sobic.006G182800 | 53778889 | 53780137 | 237 |
|  | Sobic.006G182900 | 53783513 | 53801893 | 238 |
| Chromosome 7 | Sobic.007G028600 | 2562705 | 2565651 | 239 |
|  | Sobic.007G028700 | 2567550 | 2570348 | 240 |
|  | Sobic.007G030700 | 2767986 | 2772295 | 241 |
|  | Sobic.007G032300 | 2861842 | 2863413 | 242 |
|  | Sobic.007G033400 | 2937157 | 2941121 | 243 |
|  | Sobic.007G146900 | 57708570 | 57717596 | 244 |
|  | Sobic.007G151900 | 58427906 | 58436332 | 245 |
|  | Sobic.007G153001 | 58551378 | 58554421 | 246 |
|  | Sobic.007G155300 | 58925288 | 58926097 | 247 |
|  | Sobic.007G158400 | 59271366 | 59272328 | 248 |
|  | Sobic.007G158800 | 59351417 | 59354482 | 249 |
|  | Sobic.007G160400 | 59510515 | 59514391 | 250 |
|  | Sobic.007G161800 | 59615098 | 59618876 | 251 |
|  | Sobic.007G163400 | 59785413 | 59787524 | 252 |
|  | Sobic.007G163800 | 59821905 | 59829921 | 253 |
|  | Sobic.007G164000 | 59857721 | 59861140 | 254 |
|  | Sobic.007G164200 | 59920322 | 59923651 | 255 |
|  | Sobic.007G164300 | 59934313 | 59935360 | 256 |
|  | Sobic.007G165800 | 60102559 | 60105535 | 257 |
|  | Sobic.007G166300 | 60149510 | 60153216 | 258 |
|  | Sobic.007G166900 | 60187173 | 60190004 | 259 |
|  | Sobic.007G168900 | 60374075 | 60380651 | 260 |
|  | Sobic.007G170100 | 60462488 | 60470795 | 261 |
|  | Sobic.007G170400 | 60522434 | 60524808 | 262 |
|  | Sobic.007G170500 | 60526016 | 60528195 | 263 |
|  | Sobic.007G172100 | 60704882 | 60706480 | 264 |
|  | Sobic.007G173800 | 60870267 | 60872774 | 265 |
|  | Sobic.007G175600 | 60964386 | 60964814 | 266 |
|  | Sobic.007G175701 | 60966853 | 60968967 | 267 |
|  | Sobic.007G176000 | 60979107 | 60981779 | 268 |
| Chromosome 8 | Sobic.008G035400 | 3256132 | 3257940 | 269 |
|  | Sobic.008G035600 | 3283276 | 3285600 | 270 |
|  | Sobic.008G036300 | 3382740 | 3385184 | 271 |
|  | Sobic.008G036400 | 3424236 | 3427216 | 272 |
| Chromosome 9 | Sobic.009G034300 | 3133680 | 3139601 | 273 |
|  | Sobic.009G036300 | 3314503 | 3320523 | 274 |
|  | Sobic.009G055100 | 5562293 | 5564342 | 275 |
|  | Sobic.009G056700 | 5745822 | 5755187 | 276 |
|  | Sobic.009G058600 | 6127178 | 6129463 | 277 |
|  | Sobic.009G222400 | 56518137 | 56522389 | 278 |
|  | Sobic.009G224600 | 56670457 | 56674025 | 279 |
| Chromosome 10 | Sobic.010G042400 | 3256054 | 3257914 | 280 |
|  | Sobic.010G043400 | 3373914 | 3378135 | 281 |
|  | Sobic.010G044700 | 3461212 | 3464809 | 282 |
|  | Sobic.010G227400 | 57047917 | 57050871 | 283 |
|  | Sobic.010G227800 | 57071825 | 57077620 | 284 |
