# Supplementary material for: Dissection of canopy layer-specific genetic control of leaf angle in Sorghum bicolor by RNA sequencing
Source: BMC Genomics. 2022 Feb 3;23:95. doi: 10.1186/s12864-021-08251-4 (PMC8812014; doi:10.1186/s12864-021-08251-4)
Supplement: Supplementary file 10 — Additional file 10: Supplementary Table S4. GO terms (biological process) for the 284 DEGs co-localizing with leaf angle QTL. [file 12864_2021_8251_MOESM10_ESM.docx]

**Supplementary Table S4.** GO terms (biological process) for the 284 DEGs co-localizing with leaf angle QTL.

| **Enrichment FDR** | **Genes in list** | **Total genes** | **Functional Category** | **Genes** |
| --- | --- | --- | --- | --- |
| 0.00086 | 27 | 1180 | Transmembrane transport | Sobic.002G353900 Sobic.003G050500 Sobic.003G131800 Sobic.003G133700 Sobic.006G090500 Sobic.007G176000 Sobic.010G042400 Sobic.001G157200 Sobic.001G247300 Sobic.001G349300 Sobic.001G351800 Sobic.001G379900 Sobic.001G383300 Sobic.002G352800 Sobic.003G086300 Sobic.003G096100 Sobic.003G145800 Sobic.003G185100 Sobic.004G259200 Sobic.004G314700 Sobic.005G037300 Sobic.006G042200 Sobic.007G028600 Sobic.007G153001 Sobic.007G163800 Sobic.007G166900 Sobic.009G036300 |
| 0.00164 | 34 | 1815 | Oxidation-reduction process | Sobic.001G179100 Sobic.007G166300 Sobic.001G166401 Sobic.001G167900 Sobic.001G172400 Sobic.001G250300 Sobic.001G254400 Sobic.001G274600 Sobic.001G359200 Sobic.001G359300 Sobic.001G359400 Sobic.001G363700 Sobic.001G369600 Sobic.003G035900 Sobic.003G036000 Sobic.003G036700 Sobic.003G040300 Sobic.003G152000 Sobic.003G152100 Sobic.003G152200 Sobic.003G157500 Sobic.003G188400 Sobic.003G227900 Sobic.003G404000 Sobic.004G191200 Sobic.004G315100 Sobic.005G030400 Sobic.005G042000 Sobic.006G043800 Sobic.006G048700 Sobic.006G091700 Sobic.007G033400 Sobic.007G170100 Sobic.009G055100 |
| 0.00212 | 38 | 2221 | Localization | Sobic.001G351800 Sobic.001G389300 Sobic.002G353900 Sobic.003G050500 Sobic.003G096100 Sobic.003G131800 Sobic.003G133700 Sobic.003G167500 Sobic.004G182600 Sobic.006G090500 Sobic.007G166900 Sobic.007G175600 Sobic.007G176000 Sobic.009G036300 Sobic.009G224600 Sobic.010G042400 Sobic.001G157200 Sobic.001G247300 Sobic.001G349300 Sobic.001G379900 Sobic.001G383300 Sobic.002G352800 Sobic.003G047900 Sobic.003G086300 Sobic.003G145800 Sobic.003G155600 Sobic.003G185100 Sobic.004G259200 Sobic.004G314700 Sobic.005G037300 Sobic.006G033200 Sobic.006G042200 Sobic.007G028600 Sobic.007G153001 Sobic.007G163800 Sobic.007G165800 Sobic.010G044700 Sobic.010G227800 |
| 0.00212 | 37 | 2177 | Establishment of localization | Sobic.001G351800 Sobic.001G389300 Sobic.002G353900 Sobic.003G050500 Sobic.003G096100 Sobic.003G131800 Sobic.003G133700 Sobic.003G167500 Sobic.004G182600 Sobic.006G090500 Sobic.007G166900 Sobic.007G175600 Sobic.007G176000 Sobic.009G036300 Sobic.009G224600 Sobic.010G042400 Sobic.001G157200 Sobic.001G247300 Sobic.001G349300 Sobic.001G379900 Sobic.001G383300 Sobic.002G352800 Sobic.003G047900 Sobic.003G086300 Sobic.003G145800 Sobic.003G155600 Sobic.003G185100 Sobic.004G259200 Sobic.004G314700 Sobic.005G037300 Sobic.006G033200 Sobic.006G042200 Sobic.007G028600 Sobic.007G153001 Sobic.007G163800 Sobic.010G044700 Sobic.010G227800 |
| 0.00212 | 37 | 2161 | Transport | Sobic.001G351800 Sobic.001G389300 Sobic.002G353900 Sobic.003G050500 Sobic.003G096100 Sobic.003G131800 Sobic.003G133700 Sobic.003G167500 Sobic.004G182600 Sobic.006G090500 Sobic.007G166900 Sobic.007G175600 Sobic.007G176000 Sobic.009G036300 Sobic.009G224600 Sobic.010G042400 Sobic.001G157200 Sobic.001G247300 Sobic.001G349300 Sobic.001G379900 Sobic.001G383300 Sobic.002G352800 Sobic.003G047900 Sobic.003G086300 Sobic.003G145800 Sobic.003G155600 Sobic.003G185100 Sobic.004G259200 Sobic.004G314700 Sobic.005G037300 Sobic.006G033200 Sobic.006G042200 Sobic.007G028600 Sobic.007G153001 Sobic.007G163800 Sobic.010G044700 Sobic.010G227800 |
| 0.00212 | 5 | 45 | Toxin catabolic process | Sobic.001G317600 Sobic.001G318900 Sobic.001G319100 Sobic.001G319500 Sobic.003G187100 |
| 0.00368 | 5 | 52 | Toxin metabolic process | Sobic.001G317600 Sobic.001G318900 Sobic.001G319100 Sobic.001G319500 Sobic.003G187100 |
| 0.00459 | 8 | 172 | Photosynthesis | Sobic.001G177000 Sobic.001G378100 Sobic.002G352100 Sobic.003G052500 Sobic.004G191200 Sobic.005G042000 Sobic.007G151900 Sobic.007G173800 |
| 0.01008 | 41 | 2793 | Response to stimulus | Sobic.001G156500 Sobic.001G177000 Sobic.001G317600 Sobic.001G318900 Sobic.001G319100 Sobic.001G319500 Sobic.001G389300 Sobic.003G151400 Sobic.003G157500 Sobic.003G161700 Sobic.003G187100 Sobic.004G168700 Sobic.007G176000 Sobic.001G161500 Sobic.001G172400 Sobic.001G261545 Sobic.001G344600 Sobic.001G378100 Sobic.002G353200 Sobic.003G039400 Sobic.003G051600 Sobic.003G086200 Sobic.003G096000 Sobic.003G107300 Sobic.003G121700 Sobic.003G133700 Sobic.003G149200 Sobic.003G152000 Sobic.003G152100 Sobic.003G152200 Sobic.003G185100 Sobic.003G191000 Sobic.004G178000 Sobic.004G187000 Sobic.004G216700 Sobic.005G030400 Sobic.006G042200 Sobic.006G050300 Sobic.007G163800 Sobic.007G175600 Sobic.009G055100 |
| 0.01008 | 9 | 252 | Detoxification | Sobic.001G317600 Sobic.001G318900 Sobic.001G319100 Sobic.001G319500 Sobic.003G187100 Sobic.003G152000 Sobic.003G152100 Sobic.003G152200 Sobic.009G055100 |
| 0.01258 | 7 | 159 | Regulation of hormone levels | Sobic.001G172400 Sobic.001G389300 Sobic.003G157500 Sobic.007G175600 Sobic.003G036700 Sobic.005G030400 Sobic.007G163800 |
| 0.01315 | 9 | 268 | Response to light stimulus | Sobic.001G177000 Sobic.003G157500 Sobic.001G172400 Sobic.001G261545 Sobic.003G107300 Sobic.003G121700 Sobic.003G133700 Sobic.005G030400 Sobic.007G163800 |
| 0.0135 | 9 | 272 | Response to toxic substance | Sobic.001G317600 Sobic.001G318900 Sobic.001G319100 Sobic.001G319500 Sobic.003G187100 Sobic.003G152000 Sobic.003G152100 Sobic.003G152200 Sobic.009G055100 |
| 0.01502 | 6 | 123 | Cellular modified amino acid metabolic process | Sobic.001G317600 Sobic.001G318900 Sobic.001G319100 Sobic.001G319500 Sobic.003G187100 Sobic.003G095800 |
| 0.01633 | 9 | 285 | Response to radiation | Sobic.001G177000 Sobic.003G157500 Sobic.001G172400 Sobic.001G261545 Sobic.003G107300 Sobic.003G121700 Sobic.003G133700 Sobic.005G030400 Sobic.007G163800 |
| 0.02215 | 5 | 91 | Glutathione metabolic process | Sobic.001G317600 Sobic.001G318900 Sobic.001G319100 Sobic.001G319500 Sobic.003G187100 |
| 0.02774 | 5 | 97 | Photosynthesis, light reaction | Sobic.001G177000 Sobic.002G352100 Sobic.004G191200 Sobic.007G151900 Sobic.007G173800 |
| 0.03641 | 15 | 761 | Lipid metabolic process | Sobic.001G172400 Sobic.003G150200 Sobic.003G157500 Sobic.003G162400 Sobic.003G038000 Sobic.003G087100 Sobic.003G095800 Sobic.003G114400 Sobic.003G115700 Sobic.003G178500 Sobic.003G404000 Sobic.005G030400 Sobic.006G048700 Sobic.007G158800 Sobic.007G170100 |
| 0.03641 | 14 | 682 | Ion transport | Sobic.003G133700 Sobic.003G167500 Sobic.004G182600 Sobic.006G090500 Sobic.010G042400 Sobic.001G247300 Sobic.001G379900 Sobic.003G047900 Sobic.003G086300 Sobic.003G145800 Sobic.004G314700 Sobic.007G028600 Sobic.007G153001 Sobic.010G227800 |
| 0.04363 | 12 | 552 | Cofactor metabolic process | Sobic.001G317600 Sobic.001G318900 Sobic.001G319100 Sobic.001G319500 Sobic.003G187100 Sobic.007G166300 Sobic.003G096000 Sobic.003G152000 Sobic.003G152100 Sobic.003G152200 Sobic.006G077400 Sobic.009G055100 |
| 0.04363 | 3 | 33 | Response to UV | Sobic.001G261545 Sobic.005G030400 Sobic.007G163800 |
| 0.04784 | 2 | 10 | Response to nematode | Sobic.003G133700 Sobic.007G163800 |
