# Supplementary material for: Dissection of canopy layer-specific genetic control of leaf angle in Sorghum bicolor by RNA sequencing
Source: BMC Genomics. 2022 Feb 3;23:95. doi: 10.1186/s12864-021-08251-4 (PMC8812014; doi:10.1186/s12864-021-08251-4)
Supplement: Supplementary file 11 — Additional file 11: Supplementary Table S5. Annotations for the 284 candidate DEGs co-localizing with leaf angle QTL (genes are grouped by chromosome). [file 12864_2021_8251_MOESM11_ESM.docx]

**Supplementary Table S5.** Annotations for the 284 candidate DEGs co-localizing with leaf angle QTL (genes are grouped by chromosome).

| Chromosome | **Gene Name** | **Annotation (Phytozome.net)** |
| --- | --- | --- |
| Chromosome 1 | Sobic.001G156500 | AGC_PVPK_like_kin82y.9 - ACG kinases include homologs to PKA, PKG and PKC, expressed |
|  | Sobic.001G157200 | amino acid permease family protein, putative, expressed |
|  | Sobic.001G158000 | selT/selW/selH selenoprotein domain containing protein, expressed |
|  | Sobic.001G158900 | helix-loop-helix DNA-binding domain containing protein, expressed |
|  | Sobic.001G160200 | expressed protein |
|  | Sobic.001G161500 | OsIAA12 - Auxin-responsive Aux/IAA gene family member, expressed |
|  | Sobic.001G166401 | gibberellin 20 oxidase 2, putative, expressed |
|  | Sobic.001G167900 | oxidoreductase, aldo/keto reductase family protein, putative, expressed |
|  | Sobic.001G170301 | GASR2 - Gibberellin-regulated GASA/GAST/Snakin family protein precursor, putative, expressed |
|  | Sobic.001G172400 | cytochrome P450, putative, expressed |
|  | Sobic.001G177000 | chlorophyll A-B binding protein, putative, expressed |
|  | Sobic.001G178250 | Hypothetical protein |
|  | Sobic.001G179100 | chloroplast post-illumination chlorophyll fluorescence increase protein, putative, expressed |
|  | Sobic.001G180200 | peroxisomal membrane protein, putative, expressed |
|  | Sobic.001G237700 | transposon protein, putative, CACTA, En/Spm sub-class, expressed |
|  | Sobic.001G241200 | protein kinase domain containing protein, expressed |
|  | Sobic.001G247300 | calcium-transporting ATPase, plasma membrane-type, putative, expressed |
|  | Sobic.001G250300 | oxidoreductase, putative, expressed |
|  | Sobic.001G251400 | expressed protein |
|  | Sobic.001G254400 | uncharacterized oxidoreductase, putative, expressed |
|  | Sobic.001G258200 | Ser/Thr protein phosphatase family protein, putative, expressed |
|  | Sobic.001G258300 | GDSL-like lipase/acylhydrolase, putative, expressed |
|  | Sobic.001G261545 | FAD binding domain of DNA photolyase domain containing protein, expressed |
|  | Sobic.001G261549 | POEI24 - Pollen Ole e I allergen and extensin family protein precursor, expressed |
|  | Sobic.001G263100 | endonuclease/exonuclease/phosphatase family domain containing protein, expressed |
|  | Sobic.001G267800 | thaumatin-like protein 1 precursor, putative, expressed |
|  | Sobic.001G274600 | oxidoreductase, aldo/keto reductase family protein, putative, expressed |
|  | Sobic.001G274700 | Hypothetical protein |
|  | Sobic.001G317600 | glutathione S-transferase, putative, expressed |
|  | Sobic.001G318900 | glutathione S-transferase, putative, expressed |
|  | Sobic.001G319100 | glutathione S-transferase, putative, expressed |
|  | Sobic.001G319500 | glutathione S-transferase, putative, expressed |
|  | Sobic.001G320000 | OsSub61 - Putative Subtilisin homologue, expressed |
|  | Sobic.001G323701 | zinc finger family protein, putative, expressed |
|  | Sobic.001G324700 | cysteine proteinase inhibitor 8 precursor, putative, expressed |
|  | Sobic.001G324800 | cysteine proteinase inhibitor 8 precursor, putative, expressed |
|  | Sobic.001G326200 | ZCF37, putative, expressed |
|  | Sobic.001G344600 | similar to Expressed protein |
|  | Sobic.001G349300 | similar to Putative organic cation transporter |
|  | Sobic.001G351100 | similar to Putative uncharacterized protein |
|  | Sobic.001G351200 | similar to Putative uncharacterized protein |
|  | Sobic.001G351800 | similar to Nodulin-like protein |
|  | Sobic.001G353300 | similar to Trehalose-phosphatase family protein, expressed |
|  | Sobic.001G353400 | similar to WD domain, G-beta repeat containing protein |
|  | Sobic.001G359200 | cytochrome P450 72A1, putative, expressed |
|  | Sobic.001G359300 | cytochrome P450, putative, expressed |
|  | Sobic.001G359400 | cytochrome P450, putative, expressed |
|  | Sobic.001G359700 | expressed protein |
|  | Sobic.001G363700 | cytochrome P450, putative, expressed |
|  | Sobic.001G368600 | NAD dependent epimerase/dehydratase family protein, putative, expressed |
|  | Sobic.001G368900 | IQ calmodulin-binding motif family protein, putative, expressed |
|  | Sobic.001G369401 | Hypothetical protein |
|  | Sobic.001G369600 | cytochrome P450, putative, expressed |
|  | Sobic.001G369701 | Hypothetical protein |
|  | Sobic.001G375800 | similar to Expressed protein |
|  | Sobic.001G378100 | relA-SpoT like protein RSH1, putative, expressed |
|  | Sobic.001G378300 | sucrose synthase, putative, expressed |
|  | Sobic.001G379000 | aminotransferase, putative, expressed |
|  | Sobic.001G379900 | potassium transporter, putative, expressed |
|  | Sobic.001G381500 | lung seven transmembrane domain containing protein, putative, expressed |
|  | Sobic.001G382400 | TKL_IRAK_CrRLK1L-1.5 - The CrRLK1L-1 subfamily has homology to the CrRLK1L homolog, expressed |
|  | Sobic.001G383300 | ABC transporter, ATP-binding protein, putative, expressed |
|  | Sobic.001G385900 | stress responsive protein, putative, expressed |
|  | Sobic.001G388800 | tRNA-specific adenosine deaminase 2, putative, expressed |
|  | Sobic.001G389300 | OsFBX82 - F-box domain containing protein, expressed |
|  | Sobic.001G389400 | cytochrome b5-like Heme/Steroid binding domain containing protein, expressed |
|  | Sobic.001G491900 | similar to Putative uncharacterized protein |
|  | Sobic.001G492000 | similar to Putative uncharacterized protein |
| Chromosome 2 | Sobic.002G228000 | Carboxylesterase / Procaine esterase // Tuliposide A-converting enzyme / Tuliposide-converting enzyme. |
|  | Sobic.002G228400 | MEMBER OF 'GDXG' FAMILY OF LIPOLYTIC ENZYMES // SUBFAMILY NOT NAMED |
|  | Sobic.002G351200 | similar to Putative uncharacterized protein. PFAM-Alpha/beta hydrolase family |
|  | Sobic.002G352100 | similar to LHCI-680, photosystem I antenna protein. PFAM-Chlorophyll A-B binding protein |
|  | Sobic.002G353200 | PFAM-BES1/BZR1 plant transcription factor, N-terminal |
|  | Sobic.002G353900 | similar to Os07g0582400 protein. PFAM-Sugar (and other) transporter |
|  | Sobic.002G352800 | similar to Os07g0578200 protein. PANTHER-MAJOR FACILITATOR SUPERFAMILY DOMAIN-CONTAINING PROTEIN |
| Chromosome 3 | Sobic.003G035900 | similar to Syringomycin biosynthesis enzyme-like |
|  | Sobic.003G036000 | similar to NADP-dependent malic enzyme |
|  | Sobic.003G036700 | similar to Cytokinin dehydrogenase 1 precursor |
|  | Sobic.003G037300 | PAS domain (PAS_9) |
|  | Sobic.003G038000 | phospholipase putative |
|  | Sobic.003G039400 | similar to 17.8 kDa class II heat shock protein |
|  | Sobic.003G040300 | cytochrome P450 putative |
|  | Sobic.003G042900 | UDP-glucoronosyl and UDP-glucosyl transferase domain containing protein, expressed |
|  | Sobic.003G045100 | transmembrane protein, putative, expressed |
|  | Sobic.003G045300 | Cobalt transport protein |
|  | Sobic.003G047900 | HCO3- transporter family |
|  | Sobic.003G050000 | Hypothetical protein |
|  | Sobic.003G050500 | weakly similar to Putative uncharacterized protein Os03g47200. bZIP transcription factor |
|  | Sobic.003G051600 | similar to Os01g0170600 protein |
|  | Sobic.003G052000 | similar to Putative ZmEBE-1 protein |
|  | Sobic.003G052500 | similar to Photosystem I reaction center subunit XI, chloroplast precursor |
|  | Sobic.003G079300 | Hypothetical protein |
|  | Sobic.003G081100 | similar to Os01g0136800 protein. Wall-associated receptor kinase galacturonan-binding |
|  | Sobic.003G082300 | Hsp20/alpha crystallin family |
|  | Sobic.003G082600 | similar to Os01g0135700 protein |
|  | Sobic.003G084200 | Hypothetical protein |
|  | Sobic.003G085400 | Hypothetical protein |
|  | Sobic.003G085600 | AP2 domain. |
|  | Sobic.003G086200 | NPR1 interacting (NPR1_interact) |
|  | Sobic.003G086300 | similar to Putative cation diffusion facilitator 9 |
|  | Sobic.003G087100 | ABSCISIC ACID (ABA)-DEFICIENT 4 PROTEIN (PANTHER) |
|  | Sobic.003G092500 | seven in absentia protein family protein, expressed |
|  | Sobic.003G095700 | glycosyltransferase, putative, expressed |
|  | Sobic.003G095800 | Phosphatidyl serine synthase |
|  | Sobic.003G096000 | FRUCTOSE-BISPHOSPHATE ALDOLASE 3, CHLOROPLASTIC-RELATED |
|  | Sobic.003G096100 | auxin-induced protein 5NG4, putative, expressed |
|  | Sobic.003G097100 | similar to Putative rust resistance kinase Lr10 |
|  | Sobic.003G099400 | similar to Peptidyl-prolyl cis-trans isomerase |
|  | Sobic.003G102200 | Helix-loop-helix DNA-binding domain |
|  | Sobic.003G105000 | SUGAR KINASE |
|  | Sobic.003G105500 | transferase family protein, putative, expressed |
|  | Sobic.003G107300 | similar to Light-induced protein 1-like |
|  | Sobic.003G108000 | Hypothetical protein |
|  | Sobic.003G108200 | Hypothetical protein |
|  | Sobic.003G108500 | glycosyltransferase, putative, expressed |
|  | Sobic.003G108900 | similar to Peptidase M48-like |
|  | Sobic.003G114100 | Hypothetical protein |
|  | Sobic.003G114400 | phosphatidylinositol glycan, class O (PIGO) |
|  | Sobic.003G114800 | invertase/pectin methylesterase inhibitor family protein, putative, expressed |
|  | Sobic.003G114900 | invertase/pectin methylesterase inhibitor family protein, putative, expressed |
|  | Sobic.003G115700 | lipase, putative, expressed |
|  | Sobic.003G121700 | PROTEIN PHYTOCHROME KINASE SUBSTRATE 4 |
|  | Sobic.003G131300 | similar to Os01g0277700 protein |
|  | Sobic.003G131800 | similar to Major facilitator superfamily antiporter |
|  | Sobic.003G133700 | similar to Putative solute carrier family 17 (Anion/sugar transporter), member 5 |
|  | Sobic.003G133800 | BRASSINOSTEROID INSENSITIVE 1-ASSOCIATED RECEPTOR KINASE 1-RELATED. |
|  | Sobic.003G135400 | similar to Tubulin beta-2/beta-3 chain |
|  | Sobic.003G145800 | similar to HKT8 |
|  | Sobic.003G146300 | Hypothetical protein |
|  | Sobic.003G149200 | similar to Os01g0314800 protein |
|  | Sobic.003G149500 | similar to UDP-glucuronic acid decarboxylase |
|  | Sobic.003G150200 | esterase/lipase/thioesterase family protein, putative, expressed |
|  | Sobic.003G150400 | weakly similar to Putative uncharacterized protein B1153F04.11 |
|  | Sobic.003G150600 | pectinacetylesterase domain containing protein, expressed |
|  | Sobic.003G151100 | Hypothetical protein |
|  | Sobic.003G151400 | similar to Putative PTI1-like kinase |
|  | Sobic.003G152000 | peroxidase precursor, putative, expressed |
|  | Sobic.003G152100 | peroxidase precursor, putative, expressed |
|  | Sobic.003G152200 | peroxidase precursor, putative, expressed |
|  | Sobic.003G152300 | Hypothetical protein |
|  | Sobic.003G155100 | similar to Os01g0337600 protein |
|  | Sobic.003G155600 | weakly similar to Leucine zipper protein-like |
|  | Sobic.003G157500 | similar to Putative 2-oxoglutarate-dependent dioxygenase |
|  | Sobic.003G157700 | Hypothetical protein |
|  | Sobic.003G160800 | Hypothetical protein |
|  | Sobic.003G161700 | OsWAK5 - OsWAK receptor-like protein kinase, expressed |
|  | Sobic.003G162400 | similar to Esterase/lipase/thioesterase-like protein |
|  | Sobic.003G164700 | similar to Os01g0369500 protein |
|  | Sobic.003G166200 | Hypothetical protein |
|  | Sobic.003G167500 | weakly similar to Heavy-metal-associated domain-containing protein-like |
|  | Sobic.003G173950 | Hypothetical protein |
|  | Sobic.003G174700 | similar to CLV1 receptor kinase-like protein |
|  | Sobic.003G178500 | 3-ketoacyl-CoA synthase, putative, expressed. very long chain fatty acid biosynthesis II |
|  | Sobic.003G185100 | similar to Os01g0556700 protein |
|  | Sobic.003G185400 | Hypothetical protein |
|  | Sobic.003G187100 | glutathione S-transferase, putative, expressed |
|  | Sobic.003G188400 | similar to NADP-specific glutatamate dehydrogenase, putative |
|  | Sobic.003G191000 | FAD dependent oxidoreductase domain containing protein, expressed |
|  | Sobic.003G191600 | Hypothetical protein |
|  | Sobic.003G191800 | similar to BHLH transcription-like |
|  | Sobic.003G220100 | Hypothetical protein |
|  | Sobic.003G226200 | similar to Os01g0624000 protein |
|  | Sobic.003G226300 | neutral ceramidase (ASAH2) |
|  | Sobic.003G226800 | similar to Heat shock factor RHSF13 |
|  | Sobic.003G227900 | cytochrome P450 72A1, putative, expressed |
|  | Sobic.003G232100 | Hypothetical protein |
|  | Sobic.003G233100 | PFAM:UDP-glucoronosyl and UDP-glucosyl transferase |
|  | Sobic.003G235300 | similar to Os01g0641800 protein |
|  | Sobic.003G235500 | Hypothetical protein |
|  | Sobic.003G394400 | similar to Ubiquitin-like |
|  | Sobic.003G397300 | Hypothetical protein |
|  | Sobic.003G397600 | similar to Putative uncharacterized protein |
|  | Sobic.003G397700 | similar to Leucine zipper factor-like |
|  | Sobic.003G404000 | similar to Os01g0919900 protein. PFAM-Fatty acid desaturase |
| Chromosome 4 | Sobic.004G168700 | similar to Harpin-induced protein-like |
|  | Sobic.004G170600 | GDSL-like lipase/acylhydrolase, putative, expressed |
|  | Sobic.004G176200 | similar to Inositol 5-phosphatase 3-like protein |
|  | Sobic.004G178000 | OsRR2 type-A response regulator, expressed |
|  | Sobic.004G179200 | Hypothetical protein |
|  | Sobic.004G180200 | similar to Putative uncharacterized protein |
|  | Sobic.004G182600 | weakly similar to Os06g0500300 protein |
|  | Sobic.004G185200 | similar to Homeodomain leucine zipper protein |
|  | Sobic.004G186600 | cytokinin-O-glucosyltransferase 2, putative, expressed |
|  | Sobic.004G187000 | Calmodulin binding protein-like (Calmodulin_bind) |
|  | Sobic.004G190700 | similar to Zinc finger (C3HC4-type RING finger)-like protein |
|  | Sobic.004G191200 | similar to Putative Oxygen-evolving enhancer protein 3-2, chloroplast |
|  | Sobic.004G197000 | glycine-rich cell wall structural protein precursor, putative, expressed |
|  | Sobic.004G197100 | glycine-rich cell wall structural protein precursor, putative, expressed |
|  | Sobic.004G201300 | NPH3 family |
|  | Sobic.004G207200 | Helix-loop-helix DNA-binding domain |
|  | Sobic.004G208900 | similar to Putative salt tolerance protein 3 |
|  | Sobic.004G211501 | Hypothetical protein |
|  | Sobic.004G212800 | Hypothetical protein |
|  | Sobic.004G214000 | similar to Putative adenine phosphoribosyltransferase form 2 |
|  | Sobic.004G215800 | PROTEIN PHOSPHATASE PP2A REGULATORY SUBUNIT B // SUBFAMILY NOT NAMED |
|  | Sobic.004G216700 | similar to Two-component response regulator-like PRR1 |
|  | Sobic.004G250200 | similar to RNA recognition motif (RRM)-containing protein-like |
|  | Sobic.004G258500 | similar to Os02g0720400 protein |
|  | Sobic.004G259200 | similar to ADP,ATP carrier protein 1, mitochondrial precursor |
|  | Sobic.004G314700 | similar to Putative cyclic nucleotide-binding transporter 1 |
|  | Sobic.004G314800 | Protein of unknown function (DUF3464) (DUF3464) |
|  | Sobic.004G315100 | similar to Thioredoxin-like 5 |
| Chromosome 5 | Sobic.005G030400 | cytochrome P450, putative, expressed |
|  | Sobic.005G032400 | similar to SPRY domain containing protein, expressed |
|  | Sobic.005G034000 | similar to Stem-specific protein TSJT1, putative, expressed |
|  | Sobic.005G034100 | similar to Expressed protein |
|  | Sobic.005G036600 | similar to Calmodulin-binding family protein, putative, expressed |
|  | Sobic.005G037300 | transporter, major facilitator family, putative, expressed |
|  | Sobic.005G042000 | similar to Ribulose bisphosphate carboxylase small chain, chloroplast precursor |
|  | Sobic.005G046500 | Protein tyrosine kinase |
|  | Sobic.005G047100 | Actins are highly conserved proteins that are involved in various types of cell motility and are ubiquitously expressed in all eukaryotic cells. Essential component of cell cytoskeleton; plays an important role in cytoplasmic streaming, cell shape determination, cell division, organelle movement and extension growth. |
|  | Sobic.005G126300 | BRO1-like domain |
|  | Sobic.005G129400 | similar to Expressed protein |
|  | Sobic.005G142800 | EXPORTIN 1-LIKE PROTEIN DOMAIN-CONTAINING PROTEIN |
|  | Sobic.005G142900 | similar to Expressed protein |
| Chromosome 6 | Sobic.006G028800 | interleukin-1 receptor-associated kinase 1 (IRAK1) |
|  | Sobic.006G029550 | Hypothetical protein |
|  | Sobic.006G032700 | Rhodanese-like domain |
|  | Sobic.006G033200 | similar to Probable protein transport Sec1a |
|  | Sobic.006G037700 | Phosphoribulokinase / Uridine kinase family |
|  | Sobic.006G039100 | SUBFAMILY NOT NAMED |
|  | Sobic.006G042200 | MATE efflux family protein |
|  | Sobic.006G043800 | cytochrome P450, putative, expressed |
|  | Sobic.006G045000 | SUBFAMILY NOT NAMED |
|  | Sobic.006G046000 | Hypothetical protein |
|  | Sobic.006G046600 | SUBFAMILY NOT NAMED |
|  | Sobic.006G048700 | acyl-desaturase, chloroplast precursor, putative, expressed |
|  | Sobic.006G050300 | SUBFAMILY NOT NAMED |
|  | Sobic.006G050500 | Isocitrate lyase family |
|  | Sobic.006G076000 | similar to Extensin precursor |
|  | Sobic.006G077400 | PAP_fibrillin |
|  | Sobic.006G080800 | CSLH1 - cellulose synthase-like family H, expressed |
|  | Sobic.006G086000 | No apical meristem (NAM) protein |
|  | Sobic.006G090500 | similar to Chloride channel |
|  | Sobic.006G091700 | 12-oxophytodienoate reductase, putative, expressed (JA biosynthesis) |
|  | Sobic.006G182800 | HSF-type DNA-binding |
|  | Sobic.006G182900 | Plant family of unknown function (DUF810) |
| Chromosome 7 | Sobic.007G028600 | ZINC TRANSPORT PROTEIN ZNTB. PFAM-CorA-like Mg2+ transporter protein |
|  | Sobic.007G028700 | similar to Putative uncharacterized protein. PFAM-LSD1 zinc finger |
|  | Sobic.007G030700 | similar to Putative phosphate/phosphoenolpyruvate translocator. Triose-phosphate Transporter family |
|  | Sobic.007G032300 | similar to Putative uncharacterized protein |
|  | Sobic.007G033400 | similar to Os08g0137400 protein. PFAM-Plastocyanin-like domain |
|  | Sobic.007G146900 | ARID/BRIGHT DNA binding domain |
|  | Sobic.007G151900 | PROTEIN SSU-2, ISOFORM B |
|  | Sobic.007G153001 | K+ potassium transporter |
|  | Sobic.007G155300 | similar to BHLH protein family-like |
|  | Sobic.007G158400 | Protein of unknown function, DUF617 |
|  | Sobic.007G158800 | similar to Putative uncharacterized protein: PFAM-Patatin-like phospholipase |
|  | Sobic.007G160400 | similar to Putative uncharacterized protein: PFAM-ZINC-FINGER HOMEODOMAIN PROTEIN 1 |
|  | Sobic.007G161800 | similar to PfkB type carbohydrate kinase protein family-like |
|  | Sobic.007G163400 | similar to Os08g0564700 protein: PFAM/PANTHER-Leucine Rich Repeat/KINASE-LIKE PROTEIN TMKL1-RELATED |
|  | Sobic.007G163800 | similar to PGP1: PFAM/PANTHER-ABC transporter/ABC TRANSPORTER B FAMILY MEMBER 1 |
|  | Sobic.007G164000 | similar to ATP-binding cassette transporter. PFAM/PANTHER-ABC transporter/ABC TRANSPORTER F FAMILY MEMBER 1 |
|  | Sobic.007G164200 | Protein of unknown function (DUF1618) (DUF1618) |
|  | Sobic.007G164300 | similar to Peptidyl-prolyl cis-trans isomerase |
|  | Sobic.007G165800 | similar to Putative uncharacterized protein: PFAM/PANTHER-Transferase family/BAHD ACYLTRANSFERASE DCR |
|  | Sobic.007G166300 | similar to Malate dehydrogenase [NADP], chloroplast precursor |
|  | Sobic.007G166900 | similar to Putative uncharacterized protein: PFAM/PANTHER-EamA-like transporter family/PROTEIN WALLS ARE THIN 1 |
|  | Sobic.007G168900 | similar to Kinesin heavy chain |
|  | Sobic.007G170100 | Hexadecanal dehydrogenase (acylating) / Fatty acyl-CoA reductase // Long-chain-fatty-acyl-CoA reductase / Acyl-CoA reductase // Alcohol-forming fatty acyl-CoA reductase. PFAM/PANTHER-Male sterility protein |
|  | Sobic.007G170400 | similar to Chalcone synthase 8, putative: PFAM-3-Oxoacyl-[acyl-carrier-protein (ACP)] synthase III |
|  | Sobic.007G170500 | similar to O-methyltransferase family protein |
|  | Sobic.007G172100 | similar to Putative uncharacterized protein |
|  | Sobic.007G173800 | similar to Putative uncharacterized protein |
|  | Sobic.007G175600 | similar to Putative uncharacterized protein: PFAM-Auxin responsive protein |
|  | Sobic.007G175701 | Hypothetical protein |
|  | Sobic.007G176000 | similar to Putative ripening regulated protein DDTFR18: PFAM/PANTHER-MatE/MATE EFFLUX FAMILY PROTEIN |
| Chromosome 8 | Sobic.008G035400 | Stress responsive A/B Barrel Domain |
|  | Sobic.008G035600 | similar to Flavonol 3-O-glucosyltransferase, putative, expressed/PFAM-UDP-glucoronosyl and UDP-glucosyl transferase |
|  | Sobic.008G036300 | similar to H0717B12.3 protein. PFAM-Purple acid Phosphatase, N-terminal domain |
|  | Sobic.008G036400 | Gene not annotated |
| Chromosome 9 | Sobic.009G034300 | similar to Putative uncharacterized protein OJ1127_B08.7. PFAM-Transferase family |
|  | Sobic.009G036300 | similar to Os09g0508900 protein. PFAM-Mitochondrial carrier protein |
|  | Sobic.009G055100 | similar to Peroxidase |
|  | Sobic.009G056700 | similar to Fructose-6-phosphate-2-kinase/fructose-2, 6-bisphosphatase |
|  | Sobic.009G058600 | PTHR31301:SF2 - LOB DOMAIN-CONTAINING PROTEIN 15 |
|  | Sobic.009G222400 | weakly similar to Putative uncharacterized protein |
|  | Sobic.009G224600 | similar to Os05g0553700 protein. PFAM-SNARE domain |
| Chromosome 10 | Sobic.010G042400 | SUBFAMILY NOT NAMED |
|  | Sobic.010G043400 | similar to Putative uncharacterized protein |
|  | Sobic.010G044700 | EXOCYST COMPLEX PROTEIN EXO70 // SUBFAMILY NOT NAMED |
|  | Sobic.010G227400 | ALPHA-GLUCOSIDASE // SUBFAMILY NOT NAMED. PFAM-Glycosyl hydrolases family 31 |
|  | Sobic.010G227800 | PFAM-Natural resistance-associated macrophage protein |
