# Supplementary material for: Dissection of canopy layer-specific genetic control of leaf angle in Sorghum bicolor by RNA sequencing
Source: BMC Genomics. 2022 Feb 3;23:95. doi: 10.1186/s12864-021-08251-4 (PMC8812014; doi:10.1186/s12864-021-08251-4)
Supplement: Supplementary file 13 — Additional file 13: Supplementary Table S7. Hormonal-responsive candidate genes co-localizing with leaf angle QTL on other chromosomes, and homologs in rice (Oryza sativa-Os), maize (Zea mays-Zm), and Arabidopsis (Arabidopsis thaliana-At). [file 12864_2021_8251_MOESM13_ESM.docx]

**Supplementary Table S7.** Hormonal-responsive candidate genes co-localizing with leaf angle QTL on other chromosomes, and homologs in rice (*Oryza sativa*-*Os*), maize (*Zea mays*-*Zm*) and Arabidopsis (*Arabidopsis thaliana*-*At*).

| **Sorghum gene id** | **Sorghum description** | **QTL** | ***Os***  **name** | ***Os***  **gene id** | ***Os* description** | ***Zm***  **name** | ***Zm***  **gene id** | ***Zm* description** | ***At***  **name** | ***At***  **gene id** | ***At***  **Description** |
| --- | --- | --- | --- | --- | --- | --- | --- | --- | --- | --- | --- |
| *Sobic.003G096100* | *WALLS ARE THIN 1*  EamA-like transporter family | *QLANG3.7 qP1-PFL-3.1* | Usually Multiple Acids Move In and out Transporter 1*- OsUMAMIT1* | *Os01g0117900* (E value: 1e-62, %identity: 85.60%) | Similar to nodulin-like protein | Zm00001d040089 | Zm00001d040089 | Nodulin MtN21 /EamA-like transporter family protein | *AT3G18200* | *AT3G18200* | *WAT1*-related protein |
| *Sobic.001G161500* | Auxin-responsive protein IAA12 | *QLANG1.6 QLANG1.8* | *OsIAA12* | *Os03g0633800* (E value: 6e-59, %identity: 86.57%) | Similar to IAA6 | Aux/IAA-transcription factor 22 | *Zm00001d013707* | Auxin-responsive protein *IAA4* | *SHY2* | *AT1G04240* | *AUX*/*IAA* transcriptional regulator family protein |
|  |  |  |  |  |  | Aux/IAA-transcription factor 3 | *Zm00001d033319* | Auxin-responsive protein *IAA4* | *IAA2* | *AT3G23030* | Auxin-responsive protein |
|  |  |  |  |  |  | N/A | N/A | N/A | *IAA1* | *AT4G14560* | Auxin-responsive protein |
|  |  |  |  |  |  | N/A | N/A | N/A | *IAA4* | *AT5G43700* | Auxin-responsive protein |
| *Sobic.004G178000* | Similar to Response regulator 7 | *QLANG4.1* | *OsRR2* | *Os02g0557800* (E value: 7e-66, %identity: 71.78%) | A-type response regulator, Cytokinin signaling | Cytokinin response regulator7 | Zm00001d050768 | Two-component response regulator ARR16 | N/A | N/A | N/A |
| *Sobic.001G170301* | Gibberellin regulated protein | *QLANG1.6*  *QLANG1.8* | *OsGASR2* | *Os03g0607200* (E value: 5e-46, %identity: 84.13%) | Gibberellin regulated protein family protein | Zm00001d033196 Zm00001d033200  Zm00001d033369 | Zm00001d033196 Zm00001d033200  Zm00001d033369 | Gibberellin-regulated protein 1 | N/A | N/A | N/A |
| *Sobic.002G353200* | PFAM-BES1/BZR1 plant transcription factor, N-terminal | *QLANG2.2 QLANG2.3* | *BZR1* | *Os07g0580500* (E value: *8e-114*, %identity: *81.97*%) | Transcription factor, Brassinosteroid-regulated growth response. Feedback inhibition of Brassinosteroid biosynthesis | Brassinosteroid insensitive EMS-suppressor homolog1 | *Zm00001d021927* | *BES1/BZR1* protein | *BES1 BZR1 BEH1 BEH2* | *AT1G19350 AT1G75080 AT3G50750 AT4G36780* | Brassinosteroid signaling positive regulator (BZR1) family protein  BRASSINAZOLE-RESISTANT 1  - BEH1  - BES1/BZR1 homolog 2 |
| *Sobic.003G133800* | *BRASSINOSTEROID INSENSITIVE 1-ASSOCIATED RECEPTOR KINASE 1-RELATED* | *qP1-L5-3.1 qP3-L5-3.1* | Similar to LRR protein | *Os01g0279800*  (E value: 1e-55, %identity: 92.36%) | Similar to BRASSINOSTEROID INSENSITIVE 1-associated receptor kinase 1*-OsBAK1* | BRASSINOSTEROID INSENSITIVE 1-associated receptor kinase 1 | *Zm00001d043149*  (E value-*2e-43*, %identity-*89.58*%) | BRASSINOSTEROID INSENSITIVE 1-associated receptor kinase 1 | *N/A* | *N/A* | *N/A* |
| *Sobic.001G172400* | Similar to Cytochrome P450 85A1 | *QLANG1.6 QLANG1.8* | Cytochrome P450 *85A1-brd1*  *OsDWARF* | *Os03g0602300*  (E value: *2e-92*, %identity: *83.55*)  Cytochrome P450  (E value: 5e-44, %identity: 87.97%) | Cytochrome P450 85A1 (C6-oxidase) | Brassinosteroid-deficient dwarf1 | *Zm00001d033180* | Brassinosteroid-deficient dwarf1 (BRD1) | *CYP85A1*  *CYP85A2* | *AT5G38970*  *AT3G30180* | Cytochrome P450 85A1  Cytochrome P450 85A2 |
| *Sobic.005G030400* | Cytochrome P450, family 90, subfamily A, polypeptide 1 (CYP90A1, CPD) | *QLANG5.1* | *OsCPD1* | *Os11g0143200*  (E value: 0.0, %identity: 87.64) | Similar to Cytochrome P450 90A1 | Zm00001d004957 | Zm00001d004957 | Cytochrome P450 90A1 | *CYP90A1* | *AT5G05690* | *DWF3* |
|  |  |  | *OsCPD2* | *Os12g0139300*  (E value: 0.0, %identity: 88.3) | Similar to Cytochrome P450 90A1 | Zm00001d052475 | Zm00001d052475 |  |  |  |  |
| *Sobic.001G166401* | 2OG-Fe(II) oxygenase superfamily | *QLANG1.6 QLANG1.8* | *GA20OX* | *Os03g0618300*  (E value: 3e-157, %identity: 84.00%) | Isopenicillin N synthase family protein | Gibberellin 20-oxidase4 (*GA20OX4*) | *Zm00001d013725* | Gibberellin 20-oxidase4 | *AT3G19000* | *AT3G19000* | 2-oxoglutarate (2OG) and Fe(II)-dependent oxygenase superfamily protein |
|  |  |  |  |  |  |  |  |  | *AT3G19010* | *AT3G19010* | 2-oxoglutarate (2OG) and Fe(II)-dependent oxygenase superfamily protein |
| *Sobic.001G166401* | 2OG-Fe(II) oxygenase superfamily | *QLANG1.6 QLANG1.8* | *GA20OX* | *Os03g0618300*  (E value: 3e-157, %identity: 84.00%) | Isopenicillin N synthase family protein | Gibberellin 20-oxidase4 | *Zm00001d013725* | Gibberellin 20-oxidase4 | *AT3G19000* | *AT3G19000* | 2-oxoglutarate (2OG) and Fe(II)-dependent oxygenase superfamily protein |
|  |  |  |  |  |  |  |  |  | *AT3G19010* | *AT3G19010* | 2-oxoglutarate (2OG) and Fe(II)-dependent oxygenase superfamily protein |
| *Sobic.003G036700* | Cytokinin dehydrogenase 1 | *QLANG3.1* | *OsCKX1* | *Os01g0187600*  (E value: 6e-81, %identity: 81.25%) | Similar to Cytokinin dehydrogenase 1 | Cytokinin oxidase1 | *Zm00001d039520* | Cytokinin oxidase1 | *CKX2* | *AT2G19500* | Cytokinin dehydrogenase 2 |
|  |  |  |  |  |  |  |  |  | *CKX4* | *AT4G29740* | Cytokinin dehydrogenase 4 |
|  |  |  |  |  |  |  |  |  | *CKX3* | *AT5G56970* | Cytokinin dehydrogenase 3 |
| *Sobic.006G091700* | 12-oxophytodienoate reductase (OPR) | *QLANG6.4* | N/A | N/A | N/A | Zm00001d003584 | Zm00001d003584 | 12-oxo-phytodienoic acid reductase5 | *OPR1* | *AT1G76680* | Oxophytodienoate reductase 1 |
|  |  |  |  |  |  |  |  |  | *OPR2* | *AT1G76690* | Oxophytodienoate reductase 2 |
|  |  |  |  |  |  |  |  |  | FMN-linked oxidoreductases superfamily protein | *AT1G09400* | Putative 12-oxophytodienoate reductase-like protein 1 |
|  |  |  |  |  |  |  |  |  | FMN-linked oxidoreductases superfamily protein | *AT1G17990*  *AT1G18020* | Putative 12-oxophytodienoate reductase-like protein 2B |
